# PI3K/mTORC2-RICTOR axis in early squamous non-small-cell lung cancer: genomics, molecular expression, and clinical relevance

**DOI:** 10.1177/17588359251370510

**Published:** 2025-11-07

**Authors:** Sara Pilotto, Lorenzo Belluomini, Federico Monaca, Michele Simbolo, Antonio Agostini, Andrea Mafficini, Stela Golovco, Isabella Sperduti, Emanuele Vita, Alessio Stefani, Carmine Carbone, Geny Piro, Miriam Grazia Ferrara, Filippo Lococo, Vienna Ludovini, Rita Chiari, Silvia Novello, Vincenzo Corbo, Michele Milella, Aldo Scarpa, Giampaolo Tortora, Emilio Bria

**Affiliations:** Section of Innovation Biomedicine—Oncology Area, Department of Engineering for Innovation Medicine, University of Verona School of Medicine and Verona University Hospital Trust, Verona, Italy; Section of Innovation Biomedicine—Oncology Area, Department of Engineering for Innovation Medicine, University of Verona School of Medicine and Verona University Hospital Trust, Verona, Italy; Comprehensive Cancer Center, IRCCS Fondazione Policlinico Universitario Agostino Gemelli, Rome, Italy; Dipartimento di Medicina e Chirurgia Traslazionale, Università Cattolica del Sacro Cuore, Rome, Italy; Department of Diagnostics and Public Health, University and Hospital Trust of Verona, Verona, Italy; ARC-NET Applied Research on Cancer Center, University and Hospital Trust of Verona, Verona, Italy; Comprehensive Cancer Center, IRCCS Fondazione Policlinico Universitario Agostino Gemelli, Rome, Italy; Dipartimento di Medicina e Chirurgia Traslazionale, Università Cattolica del Sacro Cuore, Rome, Italy; Department of Diagnostics and Public Health, University and Hospital Trust of Verona, Verona, Italy; ARC-NET Applied Research on Cancer Center, University and Hospital Trust of Verona, Verona, Italy; Department of Diagnostics and Public Health, University and Hospital Trust of Verona, Verona, Italy; ARC-NET Applied Research on Cancer Center, University and Hospital Trust of Verona, Verona, Italy; Biostatistics, IRCCS Regina Elena National Cancer Institute, Rome, Lazio, Italy; Comprehensive Cancer Center, IRCCS Fondazione Policlinico Universitario Agostino Gemelli, Rome, Italy; Università Cattolica del Sacro Cuore, Rome, Italy; Comprehensive Cancer Center, IRCCS Fondazione Policlinico Universitario Agostino Gemelli, Rome, Italy; Dipartimento di Medicina e Chirurgia Traslazionale, Università Cattolica del Sacro Cuore, Rome, Italy; Comprehensive Cancer Center, IRCCS Fondazione Policlinico Universitario Agostino Gemelli, Rome, Italy; Dipartimento di Medicina e Chirurgia Traslazionale, Università Cattolica del Sacro Cuore, Rome, Italy; Università Cattolica del Sacro Cuore, Rome, Italy; Thermo Fisher Scientific, Waltham, MA, USA; Comprehensive Cancer Center, IRCCS Fondazione Policlinico Universitario Agostino Gemelli, Rome, Italy; Dipartimento di Medicina e Chirurgia Traslazionale, Università Cattolica del Sacro Cuore, Rome, Italy; Division of Medical Oncology, Santa Maria della Misericordia Hospital, University of Perugia, Perugia, Italy; UOC Oncologia, AST Pesaro Urbino, Pesaro, Italy; Thoracic Oncology Unit, San Luigi Hospital, University of Turin, Orbassano, Italy; Department of Diagnostics and Public Health, University and Hospital Trust of Verona, Verona, Italy; ARC-NET Applied Research on Cancer Center, University and Hospital Trust of Verona, Verona, Italy; Section of Innovation Biomedicine—Oncology Area, Department of Engineering for Innovation Medicine, University of Verona School of Medicine and Verona University Hospital Trust, Verona, Italy; Department of Diagnostics and Public Health, University and Hospital Trust of Verona, Verona, Italy; ARC-NET Applied Research on Cancer Center, University and Hospital Trust of Verona, Verona, Italy; Comprehensive Cancer Center, IRCCS Fondazione Policlinico Universitario Agostino Gemelli, Rome, Italy; Dipartimento di Medicina e Chirurgia Traslazionale, Università Cattolica del Sacro Cuore, Rome, Italy; Comprehensive Cancer Center, IRCCS Fondazione Policlinico Universitario Agostino Gemelli, Largo Francesco Vito, 1, Rome 00168, Italy; Università Cattolica del Sacro Cuore, Rome, Italy Medical Oncology, Ospedale Isola Tiberina—Gemelli Isola, Rome, Italy

**Keywords:** lung cancer, mTORC2, next-generation sequencing, *RICTOR*, squamous

## Abstract

**Background:**

Although considerable discoveries have been made in the genomic landscape of lung adenocarcinoma, to date, little is known regarding potential prognostic factors and altered pathways in resected squamous non-small-cell lung cancer (squamous-NSCLC).

**Objective:**

We aimed to analyze the genomic background of prognostic outlier patients, selected based on a previously validated model, to assess differential genomics, and to investigate its relationship with prognosis.

**Design:**

We conducted a retrospective study on three squamous-NSCLC cohorts, integrating next-generation sequencing (NGS)-based genomic profiling and NanoString expression analysis to identify molecular alterations associated with patient prognosis.

**Methods:**

NGS analysis of somatic mutations (SM) and copy number variations (CNV) was performed by applying a 409-gene Comprehensive Cancer panel in the training set (Cohort #1) and a 56-gene customized panel in the validation set (Cohort #2). Genomic expression (NanoString) was further evaluated on an additional cohort (Cohort #3).

**Results:**

Sixty and thirty-seven (*n* = 97) Caucasian patients with available tissue out of the original 176 and 46 (*n* = 222) samples were evaluated as training and validation cohorts, respectively. CNVs were the most frequent genomic events. Molecular alterations were distributed regardless of prognosis, except for *DDR2* mutations in the good prognosis (GP) and *SMAD4* loss in the poor prognosis (PP) group. The PI3KCA/mTOR axis represented the most frequently altered pathway (42%), with *PI3KCA* mutations and *RICTOR* high gain reported only in the PP group. A genomic expression analysis performed in Cohort #3 (*n* = 35) showed that a downregulation in the PI3K/AKT/mTOR pathway was mainly evident in the GP group of patients.

**Conclusion:**

This integrated multi-step analysis identified potentially altered pathways with a biological impact on squamous-NSCLC oncogenesis, suggesting that the PI3KCA/mTOR pathway could affect the prognosis of resected SCC patients through both genomic aberrations and impaired expression.

## Introduction

Over the years, lung cancer has remained the leading cause of cancer-related deaths. Although considerable survival improvements have been achieved with the development of targeted agents in molecularly selected subsets of lung adenocarcinoma patients, their application in squamous non-small-cell lung cancer (squamous-NSCLC) is largely unsuitable, and to date, the therapeutic armamentarium for this histological subtype is limited to chemotherapy and immunotherapy, in the lack of reliable biomarkers for precision treatments.^1^ In the last decade, relevant studies have characterized squamous-NSCLC as a disease enriched in genomic and chromosomal alterations, involving also potentially targetable pathways such as PI3K/mTOR and *FGFR*.^2–5^ Nowadays, one of the emerging research strategies in cancer is centered on the study of the genomes of exceptional responders and prognostic outlier patients.^6^

Adopting this idea, we retrospectively analyzed a multicenter series of 573 surgically resected squamous-NSCLC patients and built one of the first risk classifications,^7^ which was afterward validated in a larger cohort of more than 1000 patients.^8^ This model, based on a combination of simple and easily available clinicopathological parameters (age, T-descriptor according to TNM 7th edition, lymph nodes, and grading), was able to effectively stratify resected squamous-NSCLC patients in risk classes with good prognostic accuracy. Once the best and worst prognostic performers were identified, in this study, we aimed to investigate their genomic assets using next-generation sequencing (NGS) and NanoString to identify recurrent molecular alterations or pathway dysregulations, evaluate genomic expression, and explore their association with prognosis.

## Materials and methods

### Patients

The training set (Cohort #1) comprised 60 resected Caucasian squamous-NSCLC patients with a minimum of 2 years of follow-up after removal of the primary tumor from three Italian institutions (University of Verona, Regina Elena National Cancer Institute—Rome, University of Turin), enrolled between January 2016 and December 2023. Patients were classified as good prognosis (GP) or poor prognosis (PP) in terms of disease-free survival (DFS) according to a previously published three-class prognostic model.^7,8^ A validation set (Cohort #1) included an additional cohort of 37 resected squamous-NSCLC samples provided by the University of Perugia-Italy. An extra cohort of 35 patients (Cohort #1) provided by the University Cattolica del Sacro Cuore, enrolled between January 2016 and December 2023, was used to evaluate the genomic expression through Nanostring analysis and NGS. All cases had available formalin-fixed paraffin-embedded (FFPE) tissues for molecular analysis. The reporting of this study conforms to the STROBE statement.^9^

#### Somatic mutations and copy number variations analysis by NGS

Nucleic acids extraction and qualification from FFPE samples were performed as described.^10^ Details about NGS and variant calling criteria for somatic mutations (SM) and copy number variations (CNV) are reported in the Supplemental Material.

##### Discovery screen in the training set (Cohort #1)

The Comprehensive Cancer Panel (CCP; ThermoFisher; Waltham, MA, USA) was used to investigate the mutational status of 409 genes in the training set (details on target regions are at http://www.thermofisher.com). Tumor mutational burden (TMB) was estimated according to Rizvi et al.^11^ Briefly, the number of SM identified in each sample is divided by the number of base pairs covered by CCP for a genomic space of 1.65 MB.

##### Selected analysis of the validation set (Cohort #2)

The validation set was analyzed using a custom panel targeting 56 genes (reported in the Supplemental Material). It was selected upon the results from the discovery screen using CCP, complemented with information from published exome and targeted sequencing studies.^2,12,13^

### Validation of main alterations in a different racial context using TCGA data

TCGA data of the “Lung Squamous Cell Carcinoma (TCGA PanCancer Atlas)” set, herein referred to as the TCGA set, were retrieved from cBioPortal (https://www.cbioportal.org) using both the online querying system and the R environment v4.2.2 (https://www.R-project.org/)^14^ with the cBioPortalData v1.1.1 package.^15^ Cases were selected based on the availability of genomic profiles and the following clinicopathological data: age, sex, no prior diagnosis of cancer, AJCC pathologic tumor stage, pathologic pT, pN, and pM staging information. The 409 retained cases were subset into GP (*n* = 214) and PP (*n* = 195) as above, with minor modifications to the scoring system (tumor grade information is not available for TCGA_LUSC cases). The partition of the cohort into two groups with different prognosis according to DFS and disease-specific survival was ascertained by analyzing all cases with available survival data (*n* = 340, 198 GP and 142 PP) using the Kaplan–Meier method through the survival v3.8-3 and survminer v0.5.0 R packages. Data Visualization was performed with the online cBioPortal query system, with the ComplexHeatmap v2.14.0 R package^16^ and GraphPad PRISM v6 software (GraphPad).

### Genomic expression by NanoString (Cohort #3)

FFPE samples for 35 samples (Cohort #3) of squamous-NSCLC were macrodissected after revision from the expert pathologist, and total RNA was extracted with miRNeasy FFPE Kit (Qiagen). 300 ng of total RNA were analyzed for gene expression with the Nanostring Tumor Signaling 360 panel for the nCounter MAX/FLEX System at GSTEP Immunology Facility, Fondazione Policlinico Universitario Agostino Gemelli, IRCCS. nCounter raw data (RCC files) were uploaded and analyzed on the ROSALIND platform (https://www.rosalind.bio/). Differential expression analysis (DEA) was performed by dividing the samples into two groups according to their prognosis. DEA results were used for an enrichment analysis using the R package enricher13, interrogating the 2021 Human Wikipathways library. The interactions of the top-scoring enriched genes were identified with STRING.^17^

### Statistical analysis

Descriptive statistic was used to summarize pertinent study information. One-way ANOVA, Kruskal–Wallis test, Fisher’s test with Monte Carlo simulation, and Fisher’s exact test corrected for multiple comparisons were used as appropriate. The inter-rater variability and agreement between frequencies of both SM and CNV in the training set and validation set were analyzed according to the Kappa (k) index. The index was interpreted according to the following values: <0.20 (bad); 0.21–0.40 (poor); 0.41–0.60 (moderate); 0.61–0.80 (good); and 0.81–1.00 (excellent).^18^ Correlation analysis between SM and CNV frequencies in the training set and validation set was also conducted, according to parametric (Pearson’s *r*, with 95% confidence intervals, CI) and non-parametric (Spearman’s Rho and Kendall’s Tau) coefficients; a regression equation/line was calculated according to the regression analysis (parametric R2).^19^ To visually test and weigh differences between SM and CNV frequencies in the training set and validation set, the Bland–Altman plots were determined.^19^ For all the analyses, significance was defined at the *p* < 0.05 level. The SPSS (v. 18.0), R (v. 3.2.1 and survival library v.2.38-2 for multivariate Cox regression), and MedCalc (v. 15.6) licensed statistical programs were used for all analyses. Cell line analyses, including IC50 values calculation, were performed with the GraphPad Prism 6 software.

## Results

### Patients’ cohorts

We conducted sequencing analyses on 60 resected squamous-NSCLC samples (training set), comprising 27 patients classified as GP and 33 as PP, according to a previously published three-class prognostic model.^7,8^ Training set patients’ characteristics (clinical and pathological) according to the prognostic groups are reported in Table 1. An additional cohort of 37 specimens from resected squamous-NSCLC patients, collected regardless of prognosis, was available as the validation set from the University of Perugia. The overall clinical and pathological characteristics of patients included in the training and validation sets are described in Table S1. A genomic expression analysis was performed on an extra cohort of 35 patients from the University Cattolica del Sacro Cuore. These patients were classified as GP (19 patients) and PP (16 patients) as well, according to a two-class prognostic validated model, as shown in Table S2.

**Table 1.** Clinical and pathological characteristics of the 60 patients included in the training set, separated in prognostic groups (PP and GP) according to the previously published two-class prognostic model.

Training set	PP (*N* = 33)	GP (*N* = 27)
	Patient number (%)	
Median age (years) Range	74 (67–82)	63 (43–72)
Gender		
Male	28 (84.8)	20 (74.0)
Female	5 (15.2)	7 (26.0)
Current/former smokers	30 (90.9)	23 (85.2)
Comorbidities ⩾2	19 (57.6)	8 (29.6)
ECOG PS 0–1	28 (84.8)	26 (96.3)
TNM staging (according to TNM 7th edition)		
I	0 (0.0)	14 (51.8)
II	0 (0.0)	13 (48.2)
III	33 (100.0)	0 (0.0)
Lymph nodes		
Negative	0 (0.0)	27 (100.0)
Positive	33 (100.0)	0 (0.0)
Tumor size (T descriptor according to TNM 7th edition)		
1	0 (0.0)	10 (33.3)
2	0 (0.0)	15 (59.3)
3	30 (78.8)	2 (7.4)
4	3 (21.2)	0 (0.0)
Node status (N descriptor according to TNM 7th edition)		
0	0 (0.0)	27 (100.0)
1	22 (36.4)	0 (0.0)
2	9 (51.5)	0 (0.0)
3	2 (12.1)	0 (0.0)
Grading		
1	0 (0.0)	3 (11.1)
2	5 (15.2)	20 (74.1)
3	28 (84.8)	4 (14.8)
Adjuvant therapy		
Chemotherapy (CT)	24 (72.7)	2 (7.4)
Immunotherapy after CT	1 (3.0)	0 (0.0)
No adjuvant therapy	8 (24.3)	25 (92.6)

CT, chemotherapy; ECOG PS, performance status according ECOG; GP, good prognosis; *N*, number; PP, poor prognosis.

### Molecular features

#### Training set

**Figure 1.**
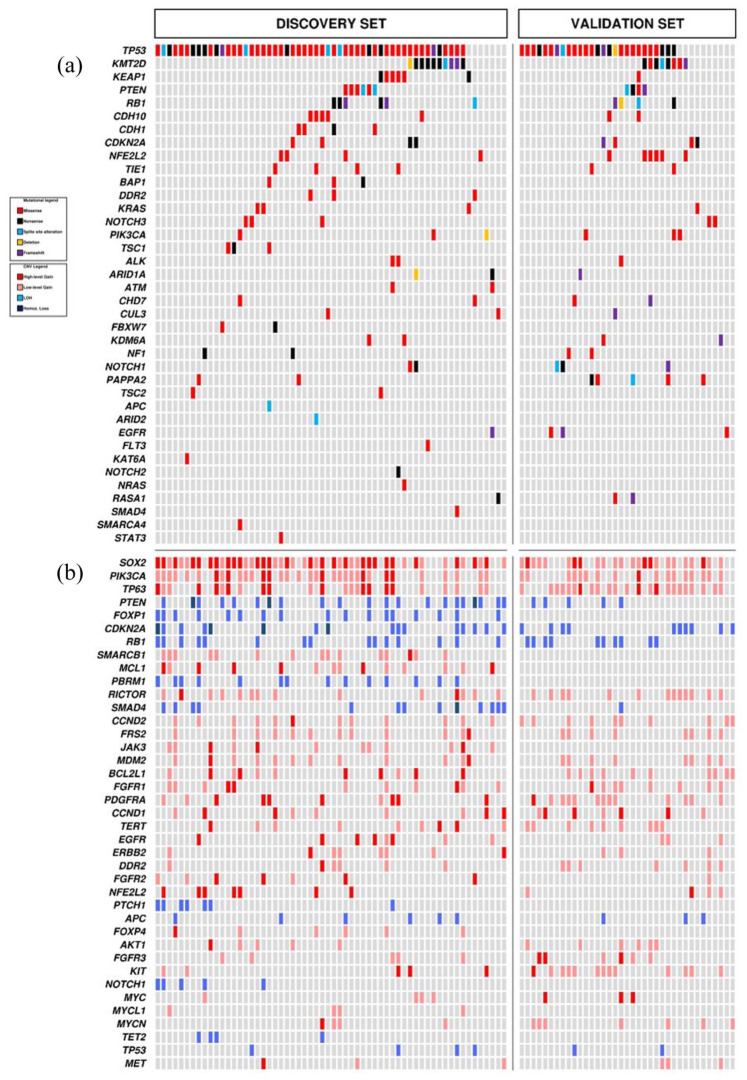
Somatic mutations (a) and CNV (b) in 60 lung squamous-NSCLC of the training set (Cohort #1) and 37 of the validation set (Cohort #2). CNV, copy number variation; LOH, loss of heterozygosity; NSCLC, non-small-cell lung cancer.

SM affecting 38 genes were detected in 59 cases (Figure 1(a) and Table 2). One mutation was observed in 9/60 cases (15.0%), more than one in 50/60 (83.3%) cases, while one case had no alterations (1.7%). The most commonly mutated genes were *TP53* (53/60; 88.3%) and *KMT2D* (10/60; 16.7%).

**Table 2.** Prevalence of SM and CNV in 60 resected squamous-NSCLC samples (training set) according to the prognostic groups (PP and GP).

SM					CNV				
Gene	PP *N* (%)	GP *N* (%)	Total *N* (%)	*p*-Value^a^	Gene	PP *N* (%)	GP *N* (%)	Total *N* (%)	*p*-Value^a^
*ALK*	1 (3.0)	1 (3.7)	2 (3.3)	*–*	*AKT1 [Gain]*	3 (9.1)	2 (7.4)	5 (8.3)	*–*
*APC*	0 (0.0)	1 (3.7)	1 (1.7)	*–*	*APC [Loss]*	3 (9.1)	3 (11.1)	6 (10.0)	*–*
*ARID1A*	1 (3.0)	1 (3.7)	2 (3.3)	*–*	*BCL2L1 [Gain]*	5 (15.2)	6 (22.2)	11 (18.3)	*–*
*ARID2*	0 (0.0)	1 (3.7)	1 (1.7)	*–*	*CCND1 [Gain]*	5 (15.2)	4 (14.8)	9 (15.0)	*–*
*ATM*	1 (3.0)	1 (3.7)	2 (3.3)	*–*	*CCND2 [Gain]*	8 (24.2)	4 (14.8)	12 (20.0)	*–*
*BAP1*	1 (3.0)	2 (7.4)	3 (5.0)	*–*	*CDKN2A [Loss]*	9 (27.3)	7 (25.9)	16 (26.7)	*–*
*CDH1*	1 (3.0)	3 (11.1)	4 (6.7)	*–*	*DDR2 [Gain]*	3 (9.1)	3 (11.1)	6 (10.0)	*–*
*CDH10*	1 (3.0)	4 (14.8)	5 (8.3)	*–*	*EGFR [Gain]*	4 (12.1)	4 (14.8)	8 (13.3)	*–*
*CDKN2A*	1 (3.0)	3 (11.1)	4 (6.7)	*–*	*ERBB2 [Gain]*	5 (15.2)	3 (11.1)	8 (13.3)	*–*
*CHD7*	1 (3.0)	1 (3.7)	2 (3.3)	*–*	*FGFR1 [Gain]*	5 (15.2)	6 (22.2)	11 (18.3)	*–*
*CUL3*	0 (0.0)	2 (7.4)	2 (3.3)	*–*	*FGFR2 [Gain]*	3 (9.1)	4 (14.8)	7 (11.7)	*–*
*DDR2*	0 (0.0)	3 (11.1)	3 (5.0)	*–*	*FGFR3 [Gain]*	2 (6.1)	3 (11.1)	5 (8.3)	*–*
*EGFR*	1 (3.0)	0 (0.0)	1 (1.7)	*–*	*FOXP1 [Loss]*	7 (21.2)	10 (37.0)	17 (28.3)	*–*
*FBXW7*	1 (3.0)	1 (3.7)	2 (3.3)	*–*	*FOXP4 [Loss]*	2 (6.1)	4 (14.8)	6 (10.0)	*–*
*FLT3*	1 (3.0)	0 (0.0)	1 (1.7)	*–*	*FRS2 [Gain]*	7 (21.2)	5 (18.5)	12 (20.0)	*–*
*KAT6A*	1 (3.0)	0 (0.0)	1 (1.7)	*–*	*JAK3 [Gain]*	6 (18.2)	6 (22.2)	12 (20.0)	*–*
*KDM6A*	1 (3.0)	1 (3.7)	2 (3.3)	*–*	*KIT [Gain]*	4 (12.1)	1 (3.7)	5 (8.3)	*–*
*KEAP1*	4 (12.1)	2 (7.4)	6 (10.0)	*–*	*MCL1 [Gain]*	9 (27.3)	5 (18.5)	14 (23.3)	*–*
*KMT2D*	7 (21.2)	3 (11.1)	10 (16.7)	*–*	*MDM2 [Gain]*	6 (18.2)	6 (22.2)	12 (20.0)	*–*
*KRAS*	1 (3.0)	2 (7.4)	3 (5.0)	*–*	*MET [Gain]*	2 (6.1)	1 (3.7)	3 (5.0)	*–*
*NF1*	2 (6.1)	0 (0.0)	2 (3.3)	*–*	*MYC [Gain]*	3 (9.1)	1 (3.7)	4 (6.7)	*–*
*NFE2L2*	1 (3.0)	3 (11.1)	4 (6.7)	*–*	*MYCL1 [Gain]*	2 (6.1)	2 (7.4)	4 (6.7)	*–*
*NOTCH1*	0 (0.0)	2 (7.4)	2 (3.3)	*–*	*MYCN [Gain]*	2 (6.1)	2 (7.4)	4 (6.7)	*–*
*NOTCH2*	1 (3.0)	0 (0.0)	1 (1.7)	*–*	*NFE2L2 [Gain]*	6 (18.2)	1 (3.7)	7 (11.7)	*–*
*NOTCH3*	1 (3.0)	2 (7.4)	3 (5.0)	*–*	*NOTCH1 [Loss]*	4 (12.1)	1 (3.7)	5 (8.3)	*–*
*NRAS*	1 (3.0)	0 (0.0)	1 (1.7)	*–*	*PBRM1 [Loss]*	7 (21.2)	7 (25.9)	14 (23.3)	*–*
*PAPPA2*	1 (3.0)	1 (3.7)	2 (3.3)	*–*	*PDGFRA [Gain]*	5 (15.2)	5 (18.5)	10 (16.7)	*–*
*PIK3CA*	3 (9.1)	0 (0.0)	3 (5.0)	*–*	*PIK3CA [Gain]*	18 (54.5)	16 (59.3)	34 (56.7)	*–*
*PTEN*	3 (9.1)	3 (11.1)	6 (10.0)	*–*	*PTCH1 [Loss]*	5 (15.2)	2 (7.4)	7 (11.7)	*–*
*RASA1*	0 (0.0)	1 (3.7)	1 (1.7)	*–*	*PTEN [Loss]*	11 (33.3)	10 (37.0)	21 (35.0)	*–*
*RB1*	2 (6.1)	4 (14.8)	6 (10.0)	*–*	*RB1 [Loss]*	9 (27.3)	6 (22.2)	15 (25.0)	*–*
*SMAD4*	1 (3.0)	0 (0.0)	1 (1.7)	*–*	*RICTOR [Gain]*	7 (21.2)	7 (25.9)	14 (23.3)	*–*
*SMARCA4*	1 (3.0)	0 (0.0)	1 (1.7)	*–*	*SMAD4 [Loss]*	11 (33.3)	2 (7.4)	13 (21.7)	*0.025*
*STAT3*	0 (0.0)	1 (3.7)	1 (1.7)	*–*	*SMARCB1 [Gain]*	7 (21.2)	8 (29.6)	15 (25.0)	*–*
*TIE1*	2 (6.1)	2 (7.4)	4 (6.7)	*–*	*SOX2 [Gain]*	24 (72.7)	23 (85.2)	47 (78.3)	*–*
*TP53*	28 (84.8)	25 (92.6)	53 (88.3)	*–*	*TERT [Gain]*	6 (18.2)	3 (11.1)	9 (15.0)	*–*
*TSC1*	1 (3.0)	2 (7.4)	3 (5.0)	*–*	*TET2 [Loss]*	1 (3.0)	3 (11.1)	4 (6.7)	*–*
*TSC2*	1 (3.0)	1 (3.7)	2 (3.3)	*–*	*TP53 [Loss]*	2 (6.1)	2 (7.4)	4 (6.7)	*–*
					*TP63 [Gain]*	14 (42.4)	10 (37.0)	24 (40.0)	*–*
					*TSC2 [Gain]*	1 (3.0)	–	1 (1.7)	*–*

a *p*-Value are reported only if <0.05 according to Fisher’s exact test.

CNV, copy number variations; GP, good prognosis; *N*, number; PP, poor prognosis; SM, somatic mutations.

CNVs were observed in all cases and involved 40 genes (Figure 1(b) and Table 2). A single CNV was observed in 3/60 cases (5.0%) and more than one in 57/60 cases (95.0%). CNV analysis showed copy number gain of *SOX2* (47/60; 78.3%) as the most frequent event, followed by the gain in *PIK3CA* (34/60; 56.7%), *TP63* (24/60; 40.0%), *SMARCB1* (15/60, 25%), *MCL1*, and *RICTOR* (each in 14/60 samples; 23.3%).

#### Validation set

**Figure 2.**
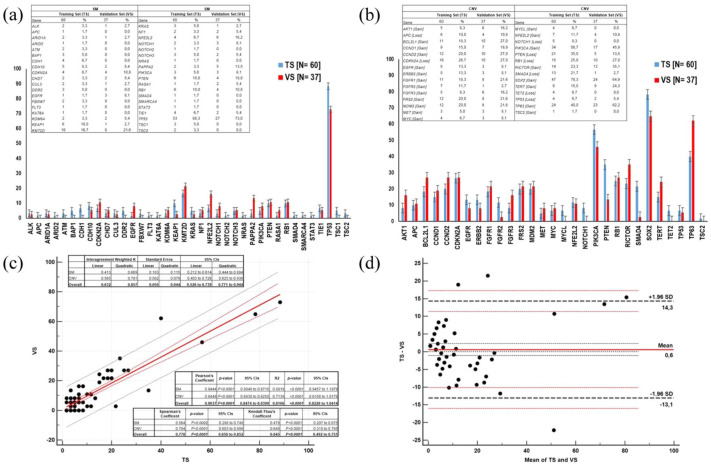
Frequencies of SM (a) and CNV (b) in training (Cohort #1) and VS (Cohort #1). Inter-agreement weighted K, correlation, and regression analysis SM and CNV frequencies between TS and VS according to Pearson, Spearman, and Kendall-Thau’s tests (c). Bland–Altman plot weighting differences in SM and CNV frequencies between TS and VS (differences plotted against TS; (d)). CI, confidence interval; CNV, copy number variation; *N*, number; SM, somatic mutation; TS, training set; VS, validation set.

An additional cohort of 37 resected squamous-NSCLC samples was used to validate the molecular analysis results of the training set (Figure 1(a) and (b)). A comparison between the two cohorts is reported in Figure 2(a) and (b). A substantial agreement for SM and CNV frequencies in the training and validation set was observed, with a significant overall correlation between them (Figure 2(c)). The Bland–Altman plot did confirm the absence of major differences or discrepancies between SM and CNV frequencies in the training and validation set (Figure 2(d)).

#### Prognostic outliers

**Figure 3.**
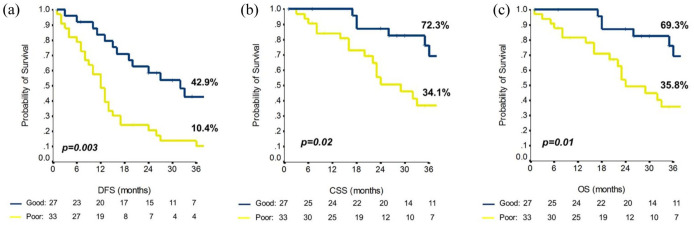
DFS (a), cancer-specific survival (b), and overall survival (c) for GP (blue line) and PP (yellow line) groups according to the published risk model. The 3-year rate for each outcome is reported, *p* value at the long-rank analysis. CSS, cancer-specific survival; DFS, disease-free survival; GP, good prognosis; OS, overall survival; PP, poor prognosis.

**Figure 4.**
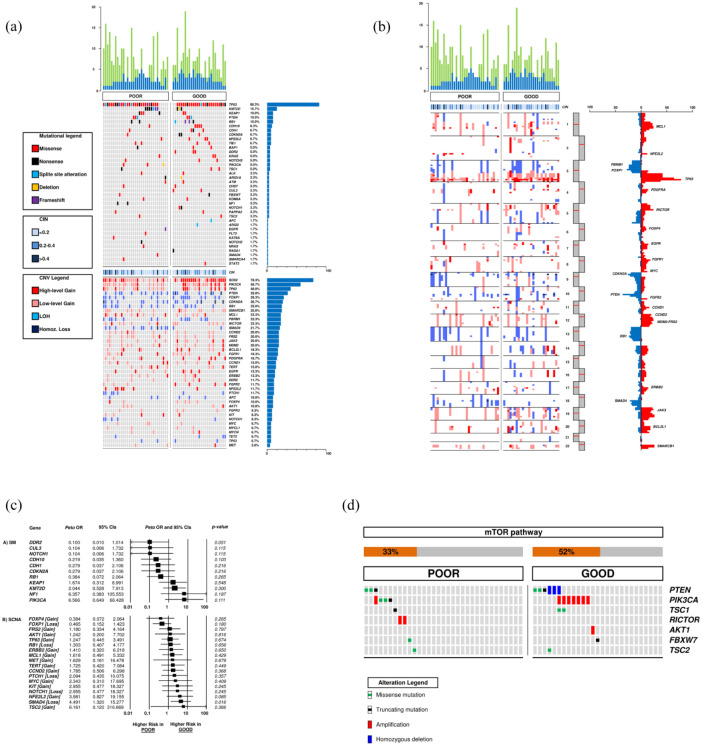
Comparison of mutational load, chromosome integrity number, SM, and CNV between PP and GP. Blue columns and green columns represent SM and CNV, respectively (a, b). OR analysis of SM and CNV according to prognosis: an OR <1 indicates a higher chance to be associated with GP; an OR >1 indicates a higher chance to be associated with PP (c). Prevalence of mTOR pathways alterations in GP and PP groups (d). CI, confidence interval; CIN, chromosome integrity number; CNV, copy number variation; GP, good prognosis; OR, odds ratio; PP, poor prognosis; SM, somatic mutation.

To characterize the molecular background of squamous-NSCLC prognostic outliers, we stratified the training cohort according to the prognostic model.^7,8^ A significant prognostic difference between patients at GP and PP was found for DFS (3-year: 42.9% and 10.4%, *p* = 0.003), cancer-specific survival (3-year: 72.3% and 34.1%, *p* = 0.02), and overall survival (3-year: 69.3% and 35.8%, *p* = 0.01; Figure 3). The prevalence of SM and CNV according to prognostic groups is reported in Figure 4(a) and (b), Table 2. Additional information is available in the Supplemental Material, and details of coverage statistics for each sample are reported in Table S3.

In the 33 cases of the PP group, *TP53* was the most frequently mutated gene (28/33; 84.8%), followed by *KMT2D* (7/33; 21.2%) and *KEAP1* (4/33; 12.1%). Interestingly, *PIK3CA* (3/33; 9.1%) and *NF1* (2/33; 6.1%) were mutated at low frequency but exclusively in the PP group. Regarding CNV, copy number gain in *SOX2* (24/33; 72.7%) was the most frequent event, followed by *PIK3CA* gain (18/33; 54.5%). *SMAD4* loss was particularly enriched in PP compared with the GP group (11/33; 33.3% vs 2/27; 7.4%, *p* = 0.025), resulting in a statistically significant higher chance to be associated with PP (OR 4.491, 95% CI 1.320–15.277, *p* = 0.016; Figure 4(c)).

In the 27 patients of the GP group, *TP53* was the most frequently mutated gene (25/27; 92.6%), followed by *CDH10*, *RB1* (4/27; 14.8%), and *NFE2L2* (3/27; 11.1%). *DDR2* mutations were exclusively identified in the GP group (3/27; 11.1%) with a higher possibility of being associated with a GP (OR 0.100, 95% CI 0.010–1.014, *p* = 0.051; Figure 4(c)). About CNV, similarly to PP, gains in *SOX2* (23/27; 85.2%) and *PIK3CA* (16/27; 59.3%). Other frequent events observed included *TP63* gain, *FOXP1*, and *PTEN* loss (10/27; 37.0%).

Although no significant difference according to the prognosis emerged in terms of alterations of the mTOR family members (detected in 42% of the training set, Figure S1), *PI3KCA* mutations and *RICTOR* high-gain amplification were detected only in the PP group (Figure 4(d)). To assess the predictive performance of the PI3K/mTORC2–RICTOR, we evaluated its classification accuracy across the training cohort (*n* = 60). The signature demonstrated a statistically significant association with PP (Fisher’s exact test, *p* < 0.0001), with a relative risk of 2.688 (95% CI: 1.823–3.963). Sensitivity and specificity were 51.5% (95% CI: 33.5%–69.2%) and 100% (95% CI: 87.2%–100%), respectively.

TMB was estimated according to Rizvi et al.^11^ using the 1.65 Mb of genomic space covered by the CCP. In the PP group, a median of 1.2 mutations/Mb (1.2 mean) was observed, while in the GP group, a median of 1.8 mutations/Mb (1.6 mean; Figure S2).

#### Comparison of molecular landscape with TCGA data

The overall landscape of altered genes in the two cohorts was compared with data retrieved from The Cancer Genome Atlas, that is, the “Lung Squamous Cell Carcinoma (TCGA PanCancer Atlas)” set, herein referred to as the TCGA set. In all, 409 cases were retrieved and categorized into GP (*n* = 214) and PP (*n* = 195) similarly to the training and validation set (Table S4). The different prognosis of the two groups was verified in 340 cases with available follow-up information by univariate analysis of disease-free (*p* = 0.0039) and disease-specific survival (*p* = 0.00036; Figure S3). Mutation frequencies showed high correlation overall (Pearson *R* = 0.942), with a similar profile of most genes and an increased prevalence of mutation of *CDH10*, *NFE2L2*, *CDKN2A*, and *PAPPA2* (Figure S4, Table S5). Copy number alterations in the TCGA set are categorized into “strong” (or “deep”) and “shallow” events and are not manually curated, as reported in the cBioPortal website (https://docs.cbioportal.org/user-guide/faq). Ideally, “strong” events should be more reliable but also more prone to false negatives. We analyzed both types of CNV in comparison with the manually curated alterations detected in cohort #1 and observed a higher degree of correlation with “strong” CNV (Pearson *R* = 0.818) compared to “shallow” CNV (Pearson *R* = 0.587; Figure S5, Table S6). There were minimal differences between GP and PP cases in the TCGA set as for mutation and CNV frequencies, and both showed a strong involvement of the mTOR pathway genes (Tables S5 and S6, Figure S6).

#### Genomic expression

Genomic expression was evaluated on an extra cohort of 35 patients, collected notwithstanding the prognosis. Based on genomic expression analysis, the GP group demonstrated a significantly higher overall survival compared to the PP group (Figure S7(A)). The DEA showed that 52 genes were downregulated in the GP group of patients, and the subsequent enrichment analysis of these genes demonstrated that these were mostly involved in the PIK3CA/AKT/mTOR pathway (Figure S7(B) and (C)). Notably, a total of 15 genes (*BAD*, *MAPK3*, *MCL1*, *AKT1S1*, *CREB1*, *MYC*, *IKBKG*, *HRAS*, *FGFR3*, *PIK3R2*, *FLT4*, *PIK3CD*, *PDGFRB*, *COL6A2*, and *LAMA4*) associated with the mTOR/PIK3CA pathway demonstrated a downregulation in patients with GP, while only the expression of *FGFR3* and *MYC* was downregulated in patients with PP (Figure S7(D)).

## Discussion

In the era of cancer molecular profiling, the design and application of risk models based on clinical parameters still provide valuable information for clinicians. Moreover, the abundance of genomic analyses did not always translate into a clinically meaningful result. Therefore, the most promising approach is likely to be represented by an integration of clinical data and genomic characterization. The results of this study support the strength of an integrative approach, extending from prognostic dichotomization and genomic analysis, able to unravel candidate aberrations with a biological impact on squamous-NSCLC oncogenesis.

The most intriguing finding we obtained concerns the validation of the PI3K/mTORC2-RICTOR axis as a crucial signaling pathway for squamous-NSCLC in terms of prevalence and differential expression in the two groups. In this regard, we observed alterations in genes involved in the mTOR pathway in 42% of the training cohort, consistent with the reported 47% by TCGA.^2^ Although after dichotomizing the patients in prognostic subgroups, no significant correlations with molecular alterations emerged, interestingly, *PI3KCA* mutations and *RICTOR* high gain were detected only in the PP group. These observations, together with emerging evidence suggesting the interpretation of the PI3K/mTOR pathway not only as a unique entity but also partitioning it into its distinct mTORC1/2-defining subunits and interactors, led our study toward a deeper investigation of the mTOR pathway.^20,21^ In contrast with *mTORC1*, *mTORC2* still represents a structure with multiple unexplored aspects that require convincing answers. Among its components, *RICTOR* has an indispensable role with constantly increasing data implicating its aberrant overexpression across numerous cancer types.^22,23^ The molecular analysis of the overall training set identified *RICTOR* copy number gain in 23.3%, similar to TCGA (16%). The identification and validation of a reliable biomarker mirroring clinically meaningful PI3K/mTOR/AKT inhibition represents a compulsory aim in lung cancer. However, clinical trials exploring the efficacy of numerous PI3K/mTOR inhibitors did not lead to solid benefit, particularly in the context of unselected patients.^21,24^

When dealing with prognosis, it should always be considered that the prognostic impact of a biomarker is not always associated with its predictive value. As an example, the case of *ERCC1* in lung cancer, where a high *ERCC1* expression is associated with improved survival but a worse outcome after cisplatin-based adjuvant chemotherapy.^25^ Focusing on the two molecular alterations that stratified according to prognosis, *DDR2* mutations were detected in 5% of our cohort, similar to what was previously described.^26^ Of interest, while to date, no correlation between *DDR2* mutations and survival has been reported,^27^ we observed a significant trend for improved survival in *DDR2* mutant patients, all belonging to the GP group. On the contrary, *SMAD4* loss was statistically significantly associated with a worse prognosis (33.3% in PP vs 7.4% in GP), and this is the first consistent evidence in lung cancer, in line with other diseases where this alteration was more widely explored.^28^ In light of the suggested increased sensitivity to DNA topoisomerase inhibitors in lung cancer, this prognostic implication is highly intriguing for further investigations.^29^

In the era of immunotherapy, TMB represents a candidate predictive biomarker. Our preliminary findings generated by NGS described a higher TMB in GP than in the PP group, contributing to enriching the prognostic speculations that are still debatable.^30–32^ In this regard, *KEAP1* mutations, a gene involved in the oxidative stress response and squamous differentiation, a predictor of immunotherapy resistance,^33^ were identified in 10.0% of the training set in agreement with TGCA.^2^

Thus, we performed a genomic expression analysis on an extra cohort of patients, collected regardless of prognosis, to assess if a differential downstream expression was present between the two prognostic groups analyzed. Interestingly, we observed a significant downregulation of the PI3KCA/mTOR pathway in patients with GP, whereas this downregulation was either absent or minimal in patients with PP.

Therefore, we might suppose that the PI3KCA/mTOR pathway could have an impact on the prognosis of resected SCC patients through both genetic aberrations and impaired genomic expression.

This study does not allow us to establish the predictive role of these alterations, and further trials are essential to address these questions.

## Conclusion

The data herein reported allowed for obtaining a representative picture of squamous-NSCLC molecular status according to patients’ prognosis and to identify altered pathways with a biological impact in oncogenesis, as the PI3K/mTORC2-RICTOR axis. Furthermore, the subsequent genomic expression analysis unveiled a downregulation of the genes involved in the PI3K/mTOR pathway in the GP group of patients, suggesting the potential association of these genes with prognosis and leading us to speculate on a differential genomic expression between groups. The results of this study support the need to identify reliable biomarkers that can enable the stratification of patients based on prognosis, thereby ensuring the implementation of a personalized approach that could potentiate the expected clinical benefit and reduce the human and economic cost resulting from a less efficacious, not-targeted treatment.

## Supplemental Material

sj-docx-1-tam-10.1177_17588359251370510 – Supplemental material for PI3K/mTORC2-RICTOR axis in early squamous non-small-cell lung cancer: genomics, molecular expression, and clinical relevance Supplemental material, sj-docx-1-tam-10.1177_17588359251370510 for PI3K/mTORC2-RICTOR axis in early squamous non-small-cell lung cancer: genomics, molecular expression, and clinical relevance by Sara Pilotto, Lorenzo Belluomini, Federico Monaca, Michele Simbolo, Antonio Agostini, Andrea Mafficini, Stela Golovco, Isabella Sperduti, Emanuele Vita, Alessio Stefani, Carmine Carbone, Geny Piro, Miriam Grazia Ferrara, Filippo Lococo, Vienna Ludovini, Rita Chiari, Silvia Novello, Vincenzo Corbo, Michele Milella, Aldo Scarpa, Giampaolo Tortora and Emilio Bria in Therapeutic Advances in Medical Oncology

sj-docx-2-tam-10.1177_17588359251370510 – Supplemental material for PI3K/mTORC2-RICTOR axis in early squamous non-small-cell lung cancer: genomics, molecular expression, and clinical relevance Supplemental material, sj-docx-2-tam-10.1177_17588359251370510 for PI3K/mTORC2-RICTOR axis in early squamous non-small-cell lung cancer: genomics, molecular expression, and clinical relevance by Sara Pilotto, Lorenzo Belluomini, Federico Monaca, Michele Simbolo, Antonio Agostini, Andrea Mafficini, Stela Golovco, Isabella Sperduti, Emanuele Vita, Alessio Stefani, Carmine Carbone, Geny Piro, Miriam Grazia Ferrara, Filippo Lococo, Vienna Ludovini, Rita Chiari, Silvia Novello, Vincenzo Corbo, Michele Milella, Aldo Scarpa, Giampaolo Tortora and Emilio Bria in Therapeutic Advances in Medical Oncology

sj-docx-3-tam-10.1177_17588359251370510 – Supplemental material for PI3K/mTORC2-RICTOR axis in early squamous non-small-cell lung cancer: genomics, molecular expression, and clinical relevance Supplemental material, sj-docx-3-tam-10.1177_17588359251370510 for PI3K/mTORC2-RICTOR axis in early squamous non-small-cell lung cancer: genomics, molecular expression, and clinical relevance by Sara Pilotto, Lorenzo Belluomini, Federico Monaca, Michele Simbolo, Antonio Agostini, Andrea Mafficini, Stela Golovco, Isabella Sperduti, Emanuele Vita, Alessio Stefani, Carmine Carbone, Geny Piro, Miriam Grazia Ferrara, Filippo Lococo, Vienna Ludovini, Rita Chiari, Silvia Novello, Vincenzo Corbo, Michele Milella, Aldo Scarpa, Giampaolo Tortora and Emilio Bria in Therapeutic Advances in Medical Oncology

sj-docx-4-tam-10.1177_17588359251370510 – Supplemental material for PI3K/mTORC2-RICTOR axis in early squamous non-small-cell lung cancer: genomics, molecular expression, and clinical relevance Supplemental material, sj-docx-4-tam-10.1177_17588359251370510 for PI3K/mTORC2-RICTOR axis in early squamous non-small-cell lung cancer: genomics, molecular expression, and clinical relevance by Sara Pilotto, Lorenzo Belluomini, Federico Monaca, Michele Simbolo, Antonio Agostini, Andrea Mafficini, Stela Golovco, Isabella Sperduti, Emanuele Vita, Alessio Stefani, Carmine Carbone, Geny Piro, Miriam Grazia Ferrara, Filippo Lococo, Vienna Ludovini, Rita Chiari, Silvia Novello, Vincenzo Corbo, Michele Milella, Aldo Scarpa, Giampaolo Tortora and Emilio Bria in Therapeutic Advances in Medical Oncology

sj-docx-5-tam-10.1177_17588359251370510 – Supplemental material for PI3K/mTORC2-RICTOR axis in early squamous non-small-cell lung cancer: genomics, molecular expression, and clinical relevance Supplemental material, sj-docx-5-tam-10.1177_17588359251370510 for PI3K/mTORC2-RICTOR axis in early squamous non-small-cell lung cancer: genomics, molecular expression, and clinical relevance by Sara Pilotto, Lorenzo Belluomini, Federico Monaca, Michele Simbolo, Antonio Agostini, Andrea Mafficini, Stela Golovco, Isabella Sperduti, Emanuele Vita, Alessio Stefani, Carmine Carbone, Geny Piro, Miriam Grazia Ferrara, Filippo Lococo, Vienna Ludovini, Rita Chiari, Silvia Novello, Vincenzo Corbo, Michele Milella, Aldo Scarpa, Giampaolo Tortora and Emilio Bria in Therapeutic Advances in Medical Oncology

sj-docx-6-tam-10.1177_17588359251370510 – Supplemental material for PI3K/mTORC2-RICTOR axis in early squamous non-small-cell lung cancer: genomics, molecular expression, and clinical relevance Supplemental material, sj-docx-6-tam-10.1177_17588359251370510 for PI3K/mTORC2-RICTOR axis in early squamous non-small-cell lung cancer: genomics, molecular expression, and clinical relevance by Sara Pilotto, Lorenzo Belluomini, Federico Monaca, Michele Simbolo, Antonio Agostini, Andrea Mafficini, Stela Golovco, Isabella Sperduti, Emanuele Vita, Alessio Stefani, Carmine Carbone, Geny Piro, Miriam Grazia Ferrara, Filippo Lococo, Vienna Ludovini, Rita Chiari, Silvia Novello, Vincenzo Corbo, Michele Milella, Aldo Scarpa, Giampaolo Tortora and Emilio Bria in Therapeutic Advances in Medical Oncology

sj-docx-7-tam-10.1177_17588359251370510 – Supplemental material for PI3K/mTORC2-RICTOR axis in early squamous non-small-cell lung cancer: genomics, molecular expression, and clinical relevance Supplemental material, sj-docx-7-tam-10.1177_17588359251370510 for PI3K/mTORC2-RICTOR axis in early squamous non-small-cell lung cancer: genomics, molecular expression, and clinical relevance by Sara Pilotto, Lorenzo Belluomini, Federico Monaca, Michele Simbolo, Antonio Agostini, Andrea Mafficini, Stela Golovco, Isabella Sperduti, Emanuele Vita, Alessio Stefani, Carmine Carbone, Geny Piro, Miriam Grazia Ferrara, Filippo Lococo, Vienna Ludovini, Rita Chiari, Silvia Novello, Vincenzo Corbo, Michele Milella, Aldo Scarpa, Giampaolo Tortora and Emilio Bria in Therapeutic Advances in Medical Oncology

sj-pdf-10-tam-10.1177_17588359251370510 – Supplemental material for PI3K/mTORC2-RICTOR axis in early squamous non-small-cell lung cancer: genomics, molecular expression, and clinical relevance Supplemental material, sj-pdf-10-tam-10.1177_17588359251370510 for PI3K/mTORC2-RICTOR axis in early squamous non-small-cell lung cancer: genomics, molecular expression, and clinical relevance by Sara Pilotto, Lorenzo Belluomini, Federico Monaca, Michele Simbolo, Antonio Agostini, Andrea Mafficini, Stela Golovco, Isabella Sperduti, Emanuele Vita, Alessio Stefani, Carmine Carbone, Geny Piro, Miriam Grazia Ferrara, Filippo Lococo, Vienna Ludovini, Rita Chiari, Silvia Novello, Vincenzo Corbo, Michele Milella, Aldo Scarpa, Giampaolo Tortora and Emilio Bria in Therapeutic Advances in Medical Oncology

sj-pdf-11-tam-10.1177_17588359251370510 – Supplemental material for PI3K/mTORC2-RICTOR axis in early squamous non-small-cell lung cancer: genomics, molecular expression, and clinical relevance Supplemental material, sj-pdf-11-tam-10.1177_17588359251370510 for PI3K/mTORC2-RICTOR axis in early squamous non-small-cell lung cancer: genomics, molecular expression, and clinical relevance by Sara Pilotto, Lorenzo Belluomini, Federico Monaca, Michele Simbolo, Antonio Agostini, Andrea Mafficini, Stela Golovco, Isabella Sperduti, Emanuele Vita, Alessio Stefani, Carmine Carbone, Geny Piro, Miriam Grazia Ferrara, Filippo Lococo, Vienna Ludovini, Rita Chiari, Silvia Novello, Vincenzo Corbo, Michele Milella, Aldo Scarpa, Giampaolo Tortora and Emilio Bria in Therapeutic Advances in Medical Oncology

sj-pdf-12-tam-10.1177_17588359251370510 – Supplemental material for PI3K/mTORC2-RICTOR axis in early squamous non-small-cell lung cancer: genomics, molecular expression, and clinical relevance Supplemental material, sj-pdf-12-tam-10.1177_17588359251370510 for PI3K/mTORC2-RICTOR axis in early squamous non-small-cell lung cancer: genomics, molecular expression, and clinical relevance by Sara Pilotto, Lorenzo Belluomini, Federico Monaca, Michele Simbolo, Antonio Agostini, Andrea Mafficini, Stela Golovco, Isabella Sperduti, Emanuele Vita, Alessio Stefani, Carmine Carbone, Geny Piro, Miriam Grazia Ferrara, Filippo Lococo, Vienna Ludovini, Rita Chiari, Silvia Novello, Vincenzo Corbo, Michele Milella, Aldo Scarpa, Giampaolo Tortora and Emilio Bria in Therapeutic Advances in Medical Oncology

sj-pdf-13-tam-10.1177_17588359251370510 – Supplemental material for PI3K/mTORC2-RICTOR axis in early squamous non-small-cell lung cancer: genomics, molecular expression, and clinical relevance Supplemental material, sj-pdf-13-tam-10.1177_17588359251370510 for PI3K/mTORC2-RICTOR axis in early squamous non-small-cell lung cancer: genomics, molecular expression, and clinical relevance by Sara Pilotto, Lorenzo Belluomini, Federico Monaca, Michele Simbolo, Antonio Agostini, Andrea Mafficini, Stela Golovco, Isabella Sperduti, Emanuele Vita, Alessio Stefani, Carmine Carbone, Geny Piro, Miriam Grazia Ferrara, Filippo Lococo, Vienna Ludovini, Rita Chiari, Silvia Novello, Vincenzo Corbo, Michele Milella, Aldo Scarpa, Giampaolo Tortora and Emilio Bria in Therapeutic Advances in Medical Oncology

sj-pdf-14-tam-10.1177_17588359251370510 – Supplemental material for PI3K/mTORC2-RICTOR axis in early squamous non-small-cell lung cancer: genomics, molecular expression, and clinical relevance Supplemental material, sj-pdf-14-tam-10.1177_17588359251370510 for PI3K/mTORC2-RICTOR axis in early squamous non-small-cell lung cancer: genomics, molecular expression, and clinical relevance by Sara Pilotto, Lorenzo Belluomini, Federico Monaca, Michele Simbolo, Antonio Agostini, Andrea Mafficini, Stela Golovco, Isabella Sperduti, Emanuele Vita, Alessio Stefani, Carmine Carbone, Geny Piro, Miriam Grazia Ferrara, Filippo Lococo, Vienna Ludovini, Rita Chiari, Silvia Novello, Vincenzo Corbo, Michele Milella, Aldo Scarpa, Giampaolo Tortora and Emilio Bria in Therapeutic Advances in Medical Oncology

sj-pdf-8-tam-10.1177_17588359251370510 – Supplemental material for PI3K/mTORC2-RICTOR axis in early squamous non-small-cell lung cancer: genomics, molecular expression, and clinical relevance Supplemental material, sj-pdf-8-tam-10.1177_17588359251370510 for PI3K/mTORC2-RICTOR axis in early squamous non-small-cell lung cancer: genomics, molecular expression, and clinical relevance by Sara Pilotto, Lorenzo Belluomini, Federico Monaca, Michele Simbolo, Antonio Agostini, Andrea Mafficini, Stela Golovco, Isabella Sperduti, Emanuele Vita, Alessio Stefani, Carmine Carbone, Geny Piro, Miriam Grazia Ferrara, Filippo Lococo, Vienna Ludovini, Rita Chiari, Silvia Novello, Vincenzo Corbo, Michele Milella, Aldo Scarpa, Giampaolo Tortora and Emilio Bria in Therapeutic Advances in Medical Oncology

sj-pdf-9-tam-10.1177_17588359251370510 – Supplemental material for PI3K/mTORC2-RICTOR axis in early squamous non-small-cell lung cancer: genomics, molecular expression, and clinical relevance Supplemental material, sj-pdf-9-tam-10.1177_17588359251370510 for PI3K/mTORC2-RICTOR axis in early squamous non-small-cell lung cancer: genomics, molecular expression, and clinical relevance by Sara Pilotto, Lorenzo Belluomini, Federico Monaca, Michele Simbolo, Antonio Agostini, Andrea Mafficini, Stela Golovco, Isabella Sperduti, Emanuele Vita, Alessio Stefani, Carmine Carbone, Geny Piro, Miriam Grazia Ferrara, Filippo Lococo, Vienna Ludovini, Rita Chiari, Silvia Novello, Vincenzo Corbo, Michele Milella, Aldo Scarpa, Giampaolo Tortora and Emilio Bria in Therapeutic Advances in Medical Oncology

## Declarations

**Ethics approval and consent to participate:** This retrospective study was conducted following the International Conference on Good Clinical Practice Standards and the ethical guidelines of the Declaration of Helsinki. The projects (AIRC-MFAG Project 14282 and AIRC-IG 20583) were approved by the local Ethics Committee.

**Consent to participate:** Written informed consent was obtained from participants.

**Consent for publication:** Not applicable.

**Availability of data and materials:** The datasets are available from the corresponding author by sending an email on reasonable request.
