# Supplementary material for: PI3K/mTORC2-RICTOR axis in early squamous non-small-cell lung cancer: genomics, molecular expression, and clinical relevance
Source: Ther Adv Med Oncol. 2025 Nov 7;17:17588359251370510. doi: 10.1177/17588359251370510 (PMC12597913; doi:10.1177/17588359251370510)
Supplement: sj-docx-1-tam-10.1177_17588359251370510 – Supplemental material for PI3K/mTORC2-RICTOR axis in early squamous non-small-cell lung cancer: genomics, molecular expression, and clinical relevance [file sj-docx-1-tam-10.1177_17588359251370510.docx]

**Supplementary Material**

**Manuscript title:**

**PI3K/mTORC2-RICTOR alterations in early squamous cell lung cancer: molecular profiling and genomic expression.**

**Supplementary Methods**

***Nucleic Acids extraction and qualification from tumor samples***

DNA was obtained from matched tumor and non-neoplastic lung included in formalin-fixed

paraffin embedded (FFPE) blocks. In particular, tumor DNA from FFPE was prepared after enrichment for neoplastic cellularity to at least 70% using manual microdissection of 10 consecutive 5-μm FFPE sections. Sections were then purified using the QIAamp DNA FFPE Tissue Kit (Qiagen) and qualified. A Tissue Macro Array (TMA) was built including all cases

(three cores each ones) and three normal lung as control.

RNA was obtained from 10 consecutive 5-μm FFPE sections using RecoverAll total nucleic acid isolation kit protocol (ThermoFisher). RNA was quantified using Qubit RNA BR Assay Kit (ThermoFisher) and qualified using Agilent RNA 6000 Nano Kit (Agilent Technologies). A RNA Integrity Number (RIN) over 5 was considered suitable for transcriptomic analysis.

**Somatic mutations (SM) and copy number variations (CNV) analysis by NGS on tumor samples**

Twenty nanograms of DNA were used for each multiplex PCR amplification. The quality of the obtained libraries was evaluated by the Agilent 2100 Bioanalyzer on-chip electrophoresis (Agilent Technologies). Emulsion PCR to construct the libraries of clonal sequences was performed with the Ion OneTouchTM OT2 System (Thermo Fisher). Sequencing was run on the Ion Proton (PI, Thermo Fisher) loaded with Ion PI Chip v2. Data analysis, including alignment to the hg19 human reference genome and variant calling, was done using the Torrent Suite Software v.5.0 (Thermo Fisher).

***Variant calling criteria for mutations and CNV***

Tumour mutations identified by Variant Caller Software v.5.0 (Thermo Fisher) were screened as follow: i) filtering-out of germline mutations identified in matched normal sample sequenced; ii) filtering-in mutations with at least 20 reads with alteration and with frequency major of 10%; iii) filtering-out artefacts through manual visualization of mutation using Integrative Genomics Viewer (IGV) v2.3. CNV detection was performed using IonReporter 5.0 pipeline (Thermo Fisher) and followed criteria: i) a Median of the Absolute values of all Pairwise Differences (MAPD) score under 1; ii) a CNV confidence number major than 20; iii) a tiles number major than 10.

***Selected analysis of validation set***

The validation set was analyzed using a custom panel targeting 56 genes: *AKT1, ALK, APC, ARID1A, ARID2, ATM, BAP1, BCL2L1, CCND1, CCND2, CDH1, CDH10, CDKN2A, CHD7, CUL3, DDR2, EGFR, ERBB2, FBXW7, FGFR1, FGFR2, FGFR3, FLT3, FRS2, KAT6A, KDM6A, KEAP1, KMT2D, KRAS, MDM2, MET, MYC, MYCL, NF1, NFE2L2, NOTCH1, NOTCH2, NOTCH3, NRAS, PAPPA2, PIK3CA, PTEN, RASA1, RB1, RICTOR, SMAD4, SMARCA4, SOX2, STAT3, TERT, TET2, TIE1, TP53, TP63, TSC1* and *TSC2*.

**Supplementary Results**

***Molecular features***

DNA from all tumour/normal paired samples was successfully amplified in multiplex PCR for the 409 genes and an adequate library for deep sequencing was obtained. The mean read length was 105 base pairs and a mean coverage of x8612 was achieved, with 75% target bases covered more than 50x. A minimum coverage of 20x was obtained in all cases. In particular sequencing yielded an average coverage of x8208 (x1654–18362) in normal samples and x9015 (x2973–14652) in tumour samples. Detail of coverage statistics for each sample has reported in **Supplementary Table S3**.
