# Supplementary material for: PI3K/mTORC2-RICTOR axis in early squamous non-small-cell lung cancer: genomics, molecular expression, and clinical relevance
Source: Ther Adv Med Oncol. 2025 Nov 7;17:17588359251370510. doi: 10.1177/17588359251370510 (PMC12597913; doi:10.1177/17588359251370510)
Supplement: sj-docx-2-tam-10.1177_17588359251370510 – Supplemental material for PI3K/mTORC2-RICTOR axis in early squamous non-small-cell lung cancer: genomics, molecular expression, and clinical relevance [file sj-docx-2-tam-10.1177_17588359251370510.docx]

**Supplementary Table S1.** Overall clinical and pathological characteristics of patients included in the training set (n = 60) and validation set (n = 37).

|  | **Training set**  **(N = 60)** | **Validation set**  **(N = 37)** |
| --- | --- | --- |
|  | Patient number (%) | |
| Median age [years]  *Range* | 68  [43 - 82] | 70  [45 - 82] |
| Gender |  |  |
| *Male* | 48 (80.0) | 30 (81.1) |
| *Female* | 12 (20.0) | 7 (18.9) |
| Current/Former Smokers | 53 (88.3) | 32 (86.5) |
| Comorbidities ≥ 2 | 27 (45.0) | 14 (37.8) |
| ECOG PS 0 - 1 | 54 (90.0) | 33 (89.2) |
| TNM Staging  [according to TNM 7th edition] |  |  |
| *I* | 14 (23.3) | 19 (51.4) |
| *II* | 13 (21.7) | 7 (18.9) |
| *III* | 33 (55.0) | 11 (29.7) |
| Lymph nodes |  |  |
| *Negative* | 27 (45.0) | 25 (67.6) |
| *Positive* | 33 (55.0) | 12 (32.4) |
| Tumor size  [T descriptor according to  TNM 7th edition] |  |  |
| *1* | 10 (16.7) | 10 (27.0) |
| *2* | 15 (25.0) | 14 (37.8) |
| *3* | 32 (53.3) | 9 (24.3) |
| *4* | 3 (5.0) | 4 (10.8) |
| Adjuvant therapy  *Chemotherapy (CT)*  *Immunotherapy after CT*  *No adjuvant therapy* | 26 (43.3)  1 (1.7)  33 (55.0) | 8 (21.6)  0 (0.0)  29 (78.4) |

**Legend -** **Supplementary Table S1**. N, number; ECOG PS, Performance Status according ECOG.
