# Supplementary material for: PI3K/mTORC2-RICTOR axis in early squamous non-small-cell lung cancer: genomics, molecular expression, and clinical relevance
Source: Ther Adv Med Oncol. 2025 Nov 7;17:17588359251370510. doi: 10.1177/17588359251370510 (PMC12597913; doi:10.1177/17588359251370510)
Supplement: sj-docx-3-tam-10.1177_17588359251370510 – Supplemental material for PI3K/mTORC2-RICTOR axis in early squamous non-small-cell lung cancer: genomics, molecular expression, and clinical relevance [file sj-docx-3-tam-10.1177_17588359251370510.docx]

**Supplementary Table S2** Clinical and pathological characteristics of the 35 patients included in the extra cohort, separated in prognostic groups (PP and GP) according to the previously published two-class prognostic model.

| **Extra Cohort** | **PP**  **(N = 16)** | **GP**  **(N = 19)** |
| --- | --- | --- |
|  | Patient number (%) | |
| Median age [years] | 68 | 74 |
| Gender |  |  |
| *Male* | 10 (62.5) | 14 (74.0) |
| *Female* | 6 (37.5) | 5 (26.0) |
| Current/Former Smokers | 14 (87.5) | 15 (78.9) |
| Comorbidities ≥ 2 | 9 (56.3) | 6 (31.6) |
| ECOG PS 0 - 1 | 14 (87.5) | 18 (94.7) |
| TNM Staging  [according to TNM 7th edition] |  |  |
| *I* | 0 (0.0) | 11 (58.0) |
| *II* | 6 (37.5) | 8 (42.0) |
| *III* | 10 (62.5) | 0 (0.0) |
| Lymph nodes |  |  |
| *Negative* | 3 (18.75) | 19 (100.0) |
| *Positive* | 13 (81.25) | 0 (0.0) |
| Tumor size  [T descriptor according to TNM 7th edition] |  |  |
| *1* | 2 (12.5) | 11 (57.9) |
| *2* | 7 (43.75) | 8 (42.1) |
| *3* | 6 (37.5) | 0 (0.0) |
| *4* | 1 (6.25) | 0 (0.0) |
| Node status  [N descriptor according to TNM 7th edition] |  |  |
| *0* | 3 (18.75) | 19 (100.0) |
| *1* | 7 (43.75) | 0 (0.0) |
| *2* | 5 (31.25) | 0 (0.0) |
| *3* | 1 (6.25) | 0 (0.0) |
| Grading |  |  |
| *1* | 0 (0.0) | 0 (0.0) |
| *2* | 0 (0.0) | 8 (42.0) |
| *3* | 16 (100.0) | 11 (58.0) |
| Adjuvant therapy  *Chemotherapy (CT)*  *Immunotherapy after CT*  *No adjuvant therapy* | 12 (75.0)  0 (0.0)  4 (25.0) | 4 (21.1)  0 (0.0)  15 (78.9) |

**Legend – Supplementary Table 2.** PP, poor prognosis; GP, good prognosis; N, number; ECOG PS, Performance Status according ECOG.
