# Supplementary material for: PI3K/mTORC2-RICTOR axis in early squamous non-small-cell lung cancer: genomics, molecular expression, and clinical relevance
Source: Ther Adv Med Oncol. 2025 Nov 7;17:17588359251370510. doi: 10.1177/17588359251370510 (PMC12597913; doi:10.1177/17588359251370510)
Supplement: sj-docx-4-tam-10.1177_17588359251370510 – Supplemental material for PI3K/mTORC2-RICTOR axis in early squamous non-small-cell lung cancer: genomics, molecular expression, and clinical relevance [file sj-docx-4-tam-10.1177_17588359251370510.docx]

| **SAMPLE** | **CHROM** | **POS** | **REF** | **ALT** | **var_freq** | **GENE** | **Feature** | **SIFT** | **PolyPhen** | **Consequence** | **HGVSc** | **HGVSp** | **EXON** | **INTRON** | **CLIN_SIG** |
| --- | --- | --- | --- | --- | --- | --- | --- | --- | --- | --- | --- | --- | --- | --- | --- |
| **1R** | chr17 | 29563006 | G | A | 10 | NF1 | XM_005257983.1 |  |  | stop_gained | XM_005257983.1:c.3941G>A | XP_005258040.1:p.Trp1314Ter | 29/59 |  |  |
| **1R** | chr17 | 7579366 | G | T | 52 | TP53 | NM_000546.5 |  |  | stop_gained | NM_000546.5:c.321C>A | NP_000537.3:p.Tyr107Ter | 4/11 |  |  |
| **2R** | chr8 | 41798785 | T | C | 43 | KAT6A | NM_001099412.1 |  | benign | missense_variant | NM_001099412.1:c.2614A>G | NP_001092882.1:p.Asn872Asp | 16/18 |  |  |
| **2R** | chr17 | 7577547 | C | T | 14 | TP53 | NM_000546.5 | 0 | probably_damaging | missense_variant | NM_000546.5:c.734G>A | NP_000537.3:p.Gly245Asp | 7/11 |  | pathogenic |
| **3R** | chr19 | 10602572 | G | A | 5 | KEAP1 | XM_005260174.1 |  |  | stop_gained | XM_005260174.1:c.1006C>T | XP_005260231.1:p.Arg336Ter | 3/6 |  |  |
| **3R** | chr13 | 48955550 | C | T | 8 | RB1 | NM_000321.2 |  |  | stop_gained | NM_000321.2:c.1666C>T | NP_000312.2:p.Arg556Ter | 17/27 |  | pathogenic |
| **3R** | chr17 | 7578419 | C | A | 40 | TP53 | NM_000546.5 |  |  | stop_gained | NM_000546.5:c.511G>T | NP_000537.3:p.Glu171Ter | 5/11 |  |  |
| **3R** | chr16 | 2134955 | C | G | 21 | TSC2 | XM_005255527.1 |  |  | missense_variant | XM_005255527.1:c.4770C>G | XP_005255584.1:p.Phe1590Leu | 35/42 |  |  |
| **4R** | chrX | 44820611 | A | G | 36 | KDM6A | XM_005272655.1 | 0 | probably_damaging | missense_variant | XM_005272655.1:c.308A>G | XP_005272712.1:p.Asn103Ser | 3/30 |  |  |
| **4R** | chr19 | 10600006 | C | T | 39 | KEAP1 | XM_005260174.1 | 0 | possibly_damaging | missense_variant | XM_005260174.1:c.1570G>A | XP_005260231.1:p.Gly524Ser | 5/6 |  |  |
| **4R** | chr1 | 115256529 | T | A | 28 | NRAS | NM_002524.4 | 0 | benign | missense_variant | NM_002524.4:c.182A>T | NP_002515.1:p.Gln61Leu | 3/7 |  |  |
| **4R** | chr17 | 7578190 | T | C | 36 | TP53 | NM_000546.5 | 0 | probably_damaging | missense_variant | NM_000546.5:c.659A>G | NP_000537.3:p.Tyr220Cys | 6/11 |  | pathogenic |
| **5R** | chr12 | 49425884 | G | A | 22 | KMT2D | XM_005269162.1 |  |  | stop_gained | XM_005269162.1:c.12604C>T | XP_005269219.1:p.Gln4202Ter | 40/55 |  |  |
| **5R** | chr3 | 178921553 | T | A | 27 | PIK3CA | NM_006218.2 | 0.01 | probably_damaging | missense_variant | NM_006218.2:c.1035T>A | NP_006209.2:p.Asn345Lys | 5/21 |  |  |
| **5R** | chr17 | 7573961 | CAGCCTGGGCATCCTTGAGTTCCA | C | 36 | TP53 | NM_000546.5 |  |  | frameshift_variant | NM_000546.5:c.1043_1065delTGGAACTCAAGGATGCCCAGGCT | NP_000537.3:p.Leu348TrpfsTer26 | 10/11 |  |  |
| **6R** | chr3 | 178928077 | TAGA | T | 5 | PIK3CA | NM_006218.2 |  |  | inframe_deletion | NM_006218.2:c.1359_1361delAGA | NP_006209.2:p.Glu453del | 8/21 |  |  |
| **7R** | chr17 | 7577548 | C | A | 63 | TP53 | NM_000546.5 | 0 | probably_damaging | missense_variant | NM_000546.5:c.733G>T | NP_000537.3:p.Gly245Cys | 7/11 |  | likely_pathogenic&pathogenic |
| **8R** | chr17 | 7577017 | A | T | 27 | TP53 | NM_000546.5 |  |  | splice_donor_variant | NM_000546.5:c.919+2T>A |  |  | 8/10 |  |
| **9R** | chr8 | 61712986 | A | G | 42 | CHD7 | NM_017780.3 |  | probably_damaging | missense_variant | NM_017780.3:c.2278A>G | NP_060250.2:p.Thr760Ala | 5/38 |  |  |
| **9R** | chr3 | 178936091 | G | A | 11 | PIK3CA | NM_006218.2 | 0.02 | probably_damaging | missense_variant | NM_006218.2:c.1633G>A | NP_006209.2:p.Glu545Lys | 10/21 |  | not_provided&pathogenic |
| **9R** | chr17 | 7577536 | T | C | 25 | TP53 | NM_000546.5 | 0 | probably_damaging | missense_variant | NM_000546.5:c.745A>G | NP_000537.3:p.Arg249Gly | 7/11 |  |  |
| **11** | chr9 | 21971101 | G | A | 44 | CDKN2A | NM_001195132.1 | 0.02 | probably_damaging | missense_variant | NM_001195132.1:c.257C>T | NP_001182061.1:p.Ala86Val | 2/4 |  |  |
| **11** | chr17 | 29683999 | C | A | 21 | NF1 | XM_005257983.1 |  |  | stop_gained | XM_005257983.1:c.7760C>A | XP_005258040.1:p.Ser2587Ter | 53/59 |  |  |
| **11** | chr19 | 11141427 | G | A | 15 | SMARCA4 | XM_005260026.1 |  |  | missense_variant | XM_005260026.1:c.3404G>A | XP_005260083.1:p.Arg1135Gln | 25/36 |  |  |
| **11** | chr17 | 7577538 | C | T | 28 | TP53 | NM_000546.5 | 0 | probably_damaging | missense_variant | NM_000546.5:c.743G>A | NP_000537.3:p.Arg248Gln | 7/11 |  | pathogenic |
| **12** | chr13 | 28608248 | C | A | 15 | FLT3 | NM_004119.2 | 0.01 | possibly_damaging | missense_variant | NM_004119.2:c.1808G>T | NP_004110.2:p.Trp603Leu | 14/24 |  |  |
| **12** | chr12 | 49433896 | G | A | 44 | KMT2D | XM_005269162.1 |  |  | stop_gained | XM_005269162.1:c.7657C>T | XP_005269219.1:p.Gln2553Ter | 32/55 |  |  |
| **12** | chr17 | 7578225 | GTC | GAC | 21 | TP53 | NM_000546.5 | 0 | probably_damaging | missense_variant | NM_000546.5:c.622_624invGAC | NP_000537.3:p.Asp208Val | 6/11 |  |  |
| **13** | chr16 | 68847364 | C | T | 11 | CDH1 | NM_004360.3 | 0.03 | probably_damaging | missense_variant | NM_004360.3:c.1286C>T | NP_004351.1:p.Pro429Leu | 9/16 |  |  |
| **13** | chr17 | 7578502 | A | G | 11 | TP53 | NM_000546.5 | 0 | probably_damaging | missense_variant | NM_000546.5:c.428T>C | NP_000537.3:p.Val143Ala | 5/11 |  |  |
| **14** | chr5 | 24488256 | A | G | 4 | CDH10 | NM_006727.3 | 0.01 | possibly_damaging | missense_variant | NM_006727.3:c.1883T>C | NP_006718.2:p.Val628Ala | 12/12 |  |  |
| **14** | chr12 | 49420844 | C | A | 28 | KMT2D | XM_005269162.1 |  |  | stop_gained | XM_005269162.1:c.14905G>T | XP_005269219.1:p.Glu4969Ter | 49/55 |  |  |
| **14** | chr17 | 7578394 | T | C | 28 | TP53 | NM_000546.5 | 0 | probably_damaging | missense_variant | NM_000546.5:c.536A>G | NP_000537.3:p.His179Arg | 5/11 |  |  |
| **15** | chr19 | 15290236 | G | T | 41 | NOTCH3 | NM_000435.2 | 0 | possibly_damaging | missense_variant | NM_000435.2:c.3399C>A | NP_000426.2:p.His1133Gln | 21/33 |  |  |
| **15** | chr17 | 7577156 | C | A | 30 | TP53 | NM_000546.5 |  |  | splice_acceptor_variant | NM_000546.5:c.783-1G>T |  |  | 7/10 |  |
| **16** | chr1 | 27088693 | GGCTCAG | GGCTGAG | 48 | ARID1A | NM_006015.4 |  |  | stop_gained | NM_006015.4:c.2302_2308delGGCTCAGinsGGCTGAG | NP_006006.3:p.Ser769Ter | 7/20 |  |  |
| **16** | chr11 | 108218071 | G | A | 20 | ATM | XM_005271562.1 | 0.02 | possibly_damaging | missense_variant | XM_005271562.1:c.8650G>A | XP_005271619.1:p.Glu2884Lys | 59/63 |  |  |
| **16** | chr7 | 55249010 | G | GACAACCCCC | 24 | EGFR | NM_005228.3 |  |  | inframe_insertion | NM_005228.3:c.2311_2319dupAACCCCCAC | NP_005219.2:p.Asn771_His773dup | 20/28 |  |  |
| **17** | chr12 | 49428260 | C | T | 90 | KMT2D | XM_005269162.1 |  |  | splice_acceptor_variant | XM_005269162.1:c.10441-1G>A |  |  | 37/54 |  |
| **17** | chr17 | 7577114 | C | A | 88 | TP53 | NM_000546.5 | 0 | probably_damaging | missense_variant | NM_000546.5:c.824G>T | NP_000537.3:p.Cys275Phe | 8/11 |  |  |
| **18** | chr12 | 49420742 | G | A | 22 | KMT2D | XM_005269162.1 |  |  | stop_gained | XM_005269162.1:c.15007C>T | XP_005269219.1:p.Gln5003Ter | 49/55 |  |  |
| **18** | chr17 | 7579377 | G | A | 30 | TP53 | NM_000546.5 |  |  | stop_gained | NM_000546.5:c.310C>T | NP_000537.3:p.Gln104Ter | 4/11 |  |  |
| **19** | chr17 | 7579372 | GCCC | GCAA | 19 | TP53 | NM_000546.5 |  |  | missense_variant | NM_000546.5:c.312_315delGGGCinsTTGC | NP_000537.3:p.GlnGly104HisCys | 4/11 |  |  |
| **19** | chr9 | 135781002 | G | A | 19 | TSC1 | XM_005272211.1 |  |  | stop_gained | XM_005272211.1:c.1963C>T | XP_005272268.1:p.Gln655Ter | 15/23 |  | not_provided |
| **20** | chr17 | 7578392 | C | A | 54 | TP53 | NM_000546.5 |  |  | stop_gained | NM_000546.5:c.538G>T | NP_000537.3:p.Glu180Ter | 5/11 |  |  |
| **21** | chr12 | 49426019 | GTTGGGGGCCCAGAAGGTTCTGGGTC | G | 17 | KMT2D | XM_005269162.1 |  |  | frameshift_variant | XM_005269162.1:c.12444_12468delGACCCAGAACCTTCTGGGCCCCCAA | XP_005269219.1:p.Met4148IlefsTer5 | 40/55 |  |  |
| **21** | chr17 | 7578457 | C | A | 24 | TP53 | NM_000546.5 | 0 | probably_damaging | missense_variant | NM_000546.5:c.473G>T | NP_000537.3:p.Arg158Leu | 5/11 |  |  |
| **22** | chr19 | 10600378 | C | A | 14 | KEAP1 | XM_005260174.1 |  |  | stop_gained | XM_005260174.1:c.1477G>T | XP_005260231.1:p.Glu493Ter | 4/6 |  |  |
| **22** | chr12 | 25398283 | ACC | AAA | 5 | KRAS | NM_033360.2 | 0 | probably_damaging | missense_variant | NM_033360.2:c.34_36delGGTinsTTT | NP_203524.1:p.Gly12Phe | 2/6 |  |  |
| **23** | chr3 | 52436860 | C | A | 12 | BAP1 | XM_005265507.1 |  |  | stop_gained | XM_005265507.1:c.1987G>T | XP_005265564.1:p.Glu663Ter | 15/17 |  |  |
| **23** | chr10 | 89711874 | G | A | 48 | PTEN | NM_000314.4 |  |  | splice_acceptor_variant | NM_000314.4:c.493-1G>A |  |  | 5/8 |  |
| **23** | chr10 | 89692911 | G | T | 22 | PTEN | NM_000314.4 | 0 | probably_damaging | missense_variant | NM_000314.4:c.395G>T | NP_000305.3:p.Gly132Val | 5/9 |  | uncertain_significance&pathogenic |
| **23** | chr17 | 7578403 | C | A | 24 | TP53 | NM_000546.5 | 0 | probably_damaging | missense_variant | NM_000546.5:c.527G>T | NP_000537.3:p.Cys176Phe | 5/11 |  |  |
| **25** | chr17 | 7577559 | G | A | 61 | TP53 | NM_000546.5 | 0 | probably_damaging | missense_variant | NM_000546.5:c.722C>T | NP_000537.3:p.Ser241Phe | 7/11 |  | likely_pathogenic&pathogenic |
| **26** | chr1 | 176668360 | C | G | 15 | PAPPA2 | XM_005245422.1 | 0 | probably_damaging | missense_variant | XM_005245422.1:c.2871C>G | XP_005245479.1:p.Phe957Leu | 8/23 |  |  |
| **26** | chr17 | 7578263 | G | A | 63 | TP53 | NM_000546.5 |  |  | stop_gained | NM_000546.5:c.586C>T | NP_000537.3:p.Arg196Ter | 6/11 |  | pathogenic |
| **27** | chr10 | 89717612 | C | T | 30 | PTEN | NM_000314.4 | 0.07 | benign | missense_variant&splice_region_variant | NM_000314.4:c.637C>T | NP_000305.3:p.Pro213Ser | 7/9 |  |  |
| **27** | chr17 | 7578518 | C | T | 31 | TP53 | NM_000546.5 | 0 | probably_damaging | missense_variant | NM_000546.5:c.412G>A | NP_000537.3:p.Ala138Thr | 5/11 |  |  |
| **28** | chr19 | 10610227 | C | A | 37 | KEAP1 | XM_005260174.1 | 0 | possibly_damaging | missense_variant | XM_005260174.1:c.483G>T | XP_005260231.1:p.Met161Ile | 2/6 |  |  |
| **28** | chr19 | 10610576 | G | A | 37 | KEAP1 | XM_005260174.1 | 0 | probably_damaging | missense_variant | XM_005260174.1:c.134C>T | XP_005260231.1:p.Ser45Phe | 2/6 |  |  |
| **28** | chr13 | 49037926 | AATCAT | AATCA | 67 | RB1 | NM_000321.2 |  |  | frameshift_variant | NM_000321.2:c.2167_2171delATCATinsATCA | NP_000312.2:p.Ile724MetfsTer2 | 21/27 |  |  |
| **28** | chr17 | 7578190 | T | C | 73 | TP53 | NM_000546.5 | 0 | probably_damaging | missense_variant | NM_000546.5:c.659A>G | NP_000537.3:p.Tyr220Cys | 6/11 |  | pathogenic |
| **29** | chr12 | 46254582 | A | T | 35 | ARID2 | NM_152641.2 |  |  | splice_acceptor_variant | NM_152641.2:c.4774-2A>T |  |  | 15/20 |  |
| **29** | chr5 | 24511474 | C | G | 24 | CDH10 | NM_006727.3 | 0.02 | benign | missense_variant | NM_006727.3:c.964G>C | NP_006718.2:p.Glu322Gln | 6/12 |  |  |
| **29** | chr1 | 43770594 | T | A | 38 | TIE1 | NM_005424.4 | 0 | benign | missense_variant | NM_005424.4:c.131T>A | NP_005415.1:p.Val44Glu | 2/23 |  |  |
| **29** | chr17 | 7576853 | C | A | 33 | TP53 | NM_000546.5 | 0.13 | benign | missense_variant&splice_region_variant | NM_000546.5:c.993G>T | NP_000537.3:p.Gln331His | 9/11 |  |  |
| **30** | chr2 | 178098944 | C | G | 22 | NFE2L2 | NM_006164.4 | 0 | probably_damaging | missense_variant | NM_006164.4:c.101G>C | NP_006155.2:p.Arg34Pro | 2/5 |  |  |
| **30** | chr17 | 7577100 | T | A | 11 | TP53 | NM_000546.5 |  |  | stop_gained | NM_000546.5:c.838A>T | NP_000537.3:p.Arg280Ter | 8/11 |  |  |
| **31** | chr4 | 153249503 | C | T | 16 | FBXW7 | NM_033632.3 |  |  | stop_gained | NM_033632.3:c.1275G>A | NP_361014.1:p.Trp425Ter | 9/12 |  |  |
| **31** | chr1 | 43777444 | C | T | 15 | TIE1 | NM_005424.4 | 0.01 | possibly_damaging | missense_variant | NM_005424.4:c.1436C>T | NP_005415.1:p.Thr479Ile | 10/23 |  |  |
| **31** | chr17 | 7578413 | C | T | 40 | TP53 | NM_000546.5 | 0.02 | probably_damaging | missense_variant | NM_000546.5:c.517G>A | NP_000537.3:p.Val173Met | 5/11 |  |  |
| **32** | chr1 | 27100070 | GGAC | GGAT | 54 | ARID1A | NM_006015.4 |  |  | splice_acceptor_variant&coding_sequence_variant | NM_006015.4:c.3867-1_3869delGGACinsGGAT |  | 16/20 | 15/19 |  |
| **32** | chr9 | 21970996 | AGCTC | AGCTA | 75 | CDKN2A | NM_001195132.1 |  |  | stop_gained | NM_001195132.1:c.358_362delGAGCTinsTAGCT | NP_001182061.1:p.Glu120Ter | 2/4 |  |  |
| **32** | chr12 | 49432129 | C | A | 36 | KMT2D | XM_005269162.1 |  |  | stop_gained | XM_005269162.1:c.9010G>T | XP_005269219.1:p.Glu3004Ter | 35/55 |  |  |
| **32** | chr9 | 139410466 | C | A | 45 | NOTCH1 | NM_017617.3 |  |  | stop_gained | NM_017617.3:c.1636G>T | NP_060087.3:p.Gly546Ter | 10/34 |  |  |
| **32** | chr17 | 7578394 | T | C | 55 | TP53 | NM_000546.5 | 0 | probably_damaging | missense_variant | NM_000546.5:c.536A>G | NP_000537.3:p.His179Arg | 5/11 |  |  |
| **33** | chr16 | 68835683 | C | T | 15 | CDH1 | NM_004360.3 | 0.2 | benign | missense_variant | NM_004360.3:c.274C>T | NP_004351.1:p.His92Tyr | 3/16 |  |  |
| **33** | chr1 | 176564448 | C | A | 11 | PAPPA2 | XM_005245422.1 | 0.28 | benign | missense_variant | XM_005245422.1:c.1708C>A | XP_005245479.1:p.His570Asn | 3/23 |  |  |
| **33** | chr17 | 7577094 | G | A | 16 | TP53 | NM_000546.5 | 0 | probably_damaging | missense_variant | NM_000546.5:c.844C>T | NP_000537.3:p.Arg282Trp | 8/11 |  | likely_benign&pathogenic |
| **34** | chrX | 44938515 | G | T | 35 | KDM6A | XM_005272655.1 | 0 | probably_damaging | missense_variant | XM_005272655.1:c.3219G>T | XP_005272712.1:p.Trp1073Cys | 21/30 |  |  |
| **34** | chr10 | 89692993 | G | C | 61 | PTEN | NM_000314.4 | 0 | probably_damaging | missense_variant | NM_000314.4:c.477G>C | NP_000305.3:p.Arg159Ser | 5/9 |  |  |
| **34** | chr17 | 7579575 | G | A | 58 | TP53 | NM_000546.5 |  |  | stop_gained | NM_000546.5:c.112C>T | NP_000537.3:p.Gln38Ter | 4/11 |  |  |
| **35** | chr2 | 178098962 | A | G | 55 | NFE2L2 | NM_006164.4 | 0 | probably_damaging | missense_variant | NM_006164.4:c.83T>C | NP_006155.2:p.Ile28Thr | 2/5 |  |  |
| **35** | chr10 | 89692791 | A | G | 74 | PTEN | NM_000314.4 | 0 | probably_damaging | missense_variant | NM_000314.4:c.275A>G | NP_000305.3:p.Asp92Gly | 5/9 |  |  |
| **35** | chr13 | 49030478 | T | TA | 62 | RB1 | NM_000321.2 |  |  | frameshift_variant | NM_000321.2:c.1959dupA | NP_000312.2:p.Val654SerfsTer14 | 19/27 |  |  |
| **35** | chr17 | 7577094 | G | C | 80 | TP53 | NM_000546.5 | 0.03 | benign | missense_variant | NM_000546.5:c.844C>G | NP_000537.3:p.Arg282Gly | 8/11 |  | likely_benign&pathogenic |
| **36** | chr12 | 49440191 | G | A | 13 | KMT2D | XM_005269162.1 |  |  | stop_gained | XM_005269162.1:c.4435C>T | XP_005269219.1:p.Gln1479Ter | 17/55 |  |  |
| **36** | chr17 | 7578266 | T | A | 21 | TP53 | NM_000546.5 | 0 | probably_damaging | missense_variant | NM_000546.5:c.583A>T | NP_000537.3:p.Ile195Phe | 6/11 |  |  |
| **37** | chr5 | 24535262 | G | T | 29 | CDH10 | NM_006727.3 | 0 | probably_damaging | missense_variant | NM_006727.3:c.773C>A | NP_006718.2:p.Thr258Lys | 5/12 |  |  |
| **37** | chr1 | 162746113 | C | T | 43 | DDR2 | XM_005245220.1 |  |  | missense_variant | XM_005245220.1:c.2281C>T | XP_005245277.1:p.Arg761Trp | 14/16 |  |  |
| **37** | chr17 | 7578398 | G | C | 45 | TP53 | NM_000546.5 | 0 | probably_damaging | missense_variant | NM_000546.5:c.532C>G | NP_000537.3:p.His178Asp | 5/11 |  |  |
| **3720** | chr13 | 49037918 | A | T | 75 | RB1 | NM_000321.2 |  |  | stop_gained | NM_000321.2:c.2158A>T | NP_000312.2:p.Lys720Ter | 21/27 |  |  |
| **3720** | chr17 | 7578556 | T | C | 67 | TP53 | NM_000546.5 |  |  | splice_acceptor_variant | NM_000546.5:c.376-2A>G |  |  | 4/10 |  |
| **3721** | chr4 | 153247256 | T | C | 52 | FBXW7 | NM_033632.3 | 0 | probably_damaging | missense_variant | NM_033632.3:c.1546A>G | NP_361014.1:p.Ser516Gly | 10/12 |  |  |
| **3721** | chr17 | 7578468 | GCC | GC | 52 | TP53 | NM_000546.5 |  |  | frameshift_variant | NM_000546.5:c.460_461delGGinsG | NP_000537.3:p.Gly154AlafsTer16 | 5/11 |  |  |
| **3722** | chr17 | 7578397 | T | G | 11 | TP53 | NM_000546.5 | 0 | probably_damaging | missense_variant | NM_000546.5:c.533A>C | NP_000537.3:p.His178Pro | 5/11 |  |  |
| **3723** | chr10 | 89717675 | C | T | 12 | PTEN | NM_000314.4 | 0.12 | benign | missense_variant | NM_000314.4:c.700C>T | NP_000305.3:p.Arg234Trp | 7/9 |  |  |
| **3723** | chr1 | 43788377 | C | T | 17 | TIE1 | NM_005424.4 | 0 | possibly_damaging | missense_variant | NM_005424.4:c.3401C>T | NP_005415.1:p.Thr1134Ile | 23/23 |  |  |
| **3723** | chr17 | 7577106 | G | A | 39 | TP53 | NM_000546.5 | 0.03 | probably_damaging | missense_variant | NM_000546.5:c.832C>T | NP_000537.3:p.Pro278Ser | 8/11 |  |  |
| **3724** |  |  |  |  |  |  |  |  |  |  |  |  |  |  |  |
| **3725** | chr12 | 49433764 | TGGGCCCCAGGGGGCTGCCCGATG | T | 100 | KMT2D | XM_005269162.1 |  |  | frameshift_variant | XM_005269162.1:c.7766_7788delCATCGGGCAGCCCCCTGGGGCCC | XP_005269219.1:p.Pro2589GlnfsTer58 | 32/55 |  |  |
| **3725** | chr18 | 48584806 | C | T | 57 | SMAD4 | NM_005359.5 | 0.59 | benign | missense_variant | NM_005359.5:c.884C>T | NP_005350.1:p.Pro295Leu | 7/12 |  |  |
| **3725** | chr17 | 7578455 | C | G | 43 | TP53 | NM_000546.5 | 0 | probably_damaging | missense_variant | NM_000546.5:c.475G>C | NP_000537.3:p.Ala159Pro | 5/11 |  | uncertain_significance |
| **3726** | chr2 | 29416570 | GAT | GAC | 100 | ALK | NM_004304.4 | 0.71 | benign | missense_variant | NM_004304.4:c.4381_4383delATCinsGTC | NP_004295.2:p.Ile1461Val | 29/29 |  |  |
| **3726** | chr19 | 10602328 | C | A | 61 | KEAP1 | XM_005260174.1 | 0 | probably_damaging | missense_variant | XM_005260174.1:c.1250G>T | XP_005260231.1:p.Gly417Val | 3/6 |  |  |
| **3726** | chr1 | 120458477 | C | A | 18 | NOTCH2 | NM_024408.3 |  |  | stop_gained | NM_024408.3:c.6868G>T | NP_077719.2:p.Glu2290Ter | 34/34 |  |  |
| **3726** | chr1 | 43777678 | G | C | 50 | TIE1 | NM_005424.4 | 0.03 | probably_damaging | missense_variant | NM_005424.4:c.1506G>C | NP_005415.1:p.Glu502Asp | 11/23 |  |  |
| **3726** | chr17 | 7577120 | C | A | 73 | TP53 | NM_000546.5 | 0 | probably_damaging | missense_variant | NM_000546.5:c.818G>T | NP_000537.3:p.Arg273Leu | 8/11 |  | pathogenic |
| **3727** | chr2 | 178096291 | G | A | 51 | NFE2L2 | NM_006164.4 | 0 | probably_damaging | missense_variant | NM_006164.4:c.1040C>T | NP_006155.2:p.Ser347Leu | 5/5 |  |  |
| **3078** | chr17 | 7577506 | C | A | 37 | TP53 | NM_000546.5 | 0.02 | probably_damaging | missense_variant | NM_000546.5:c.775G>T | NP_000537.3:p.Asp259Tyr | 7/11 |  |  |
| **3078** | chr9 | 135772704 | A | G | 7 | TSC1 | XM_005272211.1 | 0 | probably_damaging | missense_variant | XM_005272211.1:c.2842T>C | XP_005272268.1:p.Tyr948His | 22/23 |  |  |
| **3079** | chr12 | 25398284 | C | G | 11 | KRAS | NM_033360.2 | 0,02 | possibly_damaging | missense_variant | NM_033360.2:c.35G>C | NP_203524.1:p.Gly12Ala | 2/6 |  |  |
| **3079** | chr17 | 7574002 | C | G | 41 | TP53 | NM_000546.5 | 0,01 | possibly_damaging | missense_variant | NM_000546.5:c.1025G>C | NP_000537.3:p.Arg342Pro | 10/11 |  |  |
| **3080** | chr17 | 7578455 | C | G | 43 | TP53 | NM_000546.5 | 0 | probably_damaging | missense_variant | NM_000546.5:c.475G>C | NP_000537.3:p.Ala159Pro | 5/11 |  |  |
| **3081** | chr16 | 68849590 | A | C | 43 | CDH1 | NM_004360.3 | 0.07 | possibly_damaging | missense_variant | NM_004360.3:c.1493A>C | NP_004351.1:p.Asp498Ala | 10/16 |  |  |
| **3081** | chr16 | 68853241 | A | G | 16 | CDH1 | NM_004360.3 | 0.48 | benign | missense_variant | NM_004360.3:c.1624A>G | NP_004351.1:p.Ile542Val | 11/16 |  |  |
| **3081** | chr10 | 89685315 | G | A | 19 | PTEN | NM_000314.4 |  |  | splice_donor_variant | NM_000314.4:c.209+1G>A |  |  | 3/8 |  |
| **3081** | chr17 | 7577580 | T | C | 18 | TP53 | NM_000546.5 | 0 | probably_damaging | missense_variant | NM_000546.5:c.701A>G | NP_000537.3:p.Tyr234Cys | 7/11 |  |  |
| **3082** | chr17 | 7578431 | G | A | 50 | TP53 | NM_000546.5 |  |  | stop_gained | NM_000546.5:c.499C>T | NP_000537.3:p.Gln167Ter | 5/11 |  |  |
| **3083** | chr5 | 24488256 | A | G | 5 | CDH10 | NM_006727.3 | 0,01 | possibly_damaging | missense_variant | NM_006727.3:c.1883T>C | NP_006718.2:p.Val628Ala | 12/12 |  |  |
| **3083** | chr9 | 21974678 | T | C | 61 | CDKN2A | NM_001195132.1 | 0 | probably_damaging | missense_variant&splice_region_variant | NM_001195132.1:c.149A>G | NP_001182061.1:p.Gln50Arg | 1/4 |  |  |
| **3083** | chr19 | 15276244 | G | A | 15 | NOTCH3 | NM_000435.2 | 0 | benign | missense_variant | NM_000435.2:c.5750C>T | NP_000426.2:p.Ala1917Val | 31/33 |  |  |
| **3083** | chr17 | 7578406 | C | T | 58 | TP53 | NM_000546.5 | 0,11 | benign | missense_variant | NM_000546.5:c.524G>A | NP_000537.3:p.Arg175His | 5/11 |  | pathogenic |
| **3084** | chr5 | 112136975 | G | A | 5 | APC | XM_005271975.1 |  |  | splice_acceptor_variant | XM_005271975.1:c.730-1G>A |  |  | 7/15 |  |
| **3084** | chr3 | 52436907 | C | T | 5 | BAP1 | XM_005265507.1 |  |  | missense_variant | XM_005265507.1:c.1940G>A | XP_005265564.1:p.Cys647Tyr | 15/17 |  |  |
| **3084** | chr17 | 7577082 | C | T | 39 | TP53 | NM_000546.5 | 0 | probably_damaging | missense_variant | NM_000546.5:c.856G>A | NP_000537.3:p.Glu286Lys | 8/11 |  |  |
| **3084** | chr9 | 135787690 | C | T | 5 | TSC1 | XM_005272211.1 | 0.55 | benign | missense_variant | XM_005272211.1:c.892G>A | XP_005272268.1:p.Ala298Thr | 9/23 |  |  |
| **3085** | chr2 | 29432687 | C | G | 36 | ALK | NM_004304.4 | 0 | probably_damaging | missense_variant | NM_004304.4:c.3801G>C | NP_004295.2:p.Lys1267Asn | 25/29 |  |  |
| **3085** | chr11 | 108158393 | C | A | 44 | ATM | XM_005271562.1 |  | benign | missense_variant | XM_005271562.1:c.4060C>A | XP_005271619.1:p.Pro1354Thr | 27/63 |  | uncertain_significance |
| **3085** | chr19 | 10600444 | G | C | 16 | KEAP1 | XM_005260174.1 | 0,09 | benign | missense_variant | XM_005260174.1:c.1411C>G | XP_005260231.1:p.Leu471Val | 4/6 |  |  |
| **3085** | chr17 | 7577575 | A | G | 66 | TP53 | NM_000546.5 | 0 | probably_damaging | missense_variant | NM_000546.5:c.706T>C | NP_000537.3:p.Tyr236His | 7/11 |  |  |
| **3086** | chr5 | 24492991 | C | G | 21 | CDH10 | NM_006727.3 | 0,78 | possibly_damaging | missense_variant | NM_006727.3:c.1559G>C | NP_006718.2:p.Gly520Ala | 10/12 |  |  |
| **3086** | chr2 | 225342997 | T | C | 55 | CUL3 | NM_001257198.1 |  |  | missense_variant | NM_001257198.1:c.2113A>G | NP_001244127.1:p.Arg705Gly | 15/16 |  |  |
| **3086** | chr17 | 7578369 | A | G | 38 | TP53 | NM_000546.5 |  |  | splice_donor_variant | NM_000546.5:c.559+2T>C |  |  | 5/10 |  |
| **3087** | chr9 | 21970901 | C | A | 59 | CDKN2A | NM_001195132.1 |  |  | stop_gained&splice_region_variant | NM_001195132.1:c.457G>T | NP_001182061.1:p.Glu153Ter | 2/4 |  |  |
| **3087** | chr12 | 49427729 | CCTT | C | 21 | KMT2D | XM_005269162.1 |  |  | inframe_deletion | XM_005269162.1:c.10756_10758delAAG | XP_005269219.1:p.Lys3586del | 40/55 |  |  |
| **3087** | chr9 | 139393360 | C | A | 58 | NOTCH1 | NM_017617.3 | 0 | probably_damaging | missense_variant | NM_017617.3:c.6171G>T | NP_060087.3:p.Gln2057His | 33/34 |  |  |
| **3087** | chr17 | 7577559 | G | A | 84 | TP53 | NM_000546.5 | 0 | probably_damaging | missense_variant | NM_000546.5:c.722C>T | NP_000537.3:p.Ser241Phe | 7/11 |  | likely_pathogenic&pathogenic |
| **3088** | chr2 | 225400306 | T | C | 12 | CUL3 | NM_001257198.1 |  |  | missense_variant | NM_001257198.1:c.335A>G | NP_001244127.1:p.Asn112Ser | 3/16 |  |  |
| **3088** | chr5 | 86648999 | C | T | 14 | RASA1 | NM_002890.2 |  |  | stop_gained | NM_002890.2:c.1279C>T | NP_002881.1:p.Arg427Ter | 9/25 |  |  |
| **3089** | chr3 | 52437161 | G | A | 46 | BAP1 | XM_005265507.1 |  |  | missense_variant | XM_005265507.1:c.1883C>T | XP_005265564.1:p.Ser628Leu | 14/17 |  |  |
| **3089** | chr16 | 68849604 | C | T | 33 | CDH1 | NM_004360.3 |  |  | stop_gained | NM_004360.3:c.1507C>T | NP_004351.1:p.Gln503Ter | 10/16 |  |  |
| **3089** | chr1 | 162748409 | G | A | 40 | DDR2 | XM_005245220.1 |  |  | missense_variant | XM_005245220.1:c.2368G>A | XP_005245277.1:p.Val790Ile | 15/16 |  |  |
| **3089** | chr13 | 48955432 | G | A | 34 | RB1 | NM_000321.2 |  |  | stop_gained | NM_000321.2:c.1548G>A | NP_000312.2:p.Trp516Ter | 17/27 |  |  |
| **3089** | chr17 | 7578469 | C | A | 51 | TP53 | NM_000546.5 | 0 | probably_damaging | missense_variant | NM_000546.5:c.461G>T | NP_000537.3:p.Gly154Val | 5/11 |  |  |
| **3090** | chr12 | 25398285 | C | A | 45 | KRAS | NM_033360.2 | 0,04 | probably_damaging | missense_variant | NM_033360.2:c.34G>T | NP_203524.1:p.Gly12Cys | 2/6 |  |  |
| **3090** | chr17 | 7578534 | C | G | 36 | TP53 | NM_000546.5 | 0 | probably_damaging | missense_variant | NM_000546.5:c.396G>C | NP_000537.3:p.Lys132Asn | 5/11 |  |  |
| **3091** | chr2 | 178098810 | C | G | 26 | NFE2L2 | NM_006164.4 | 0 | probably_damaging | missense_variant | NM_006164.4:c.235G>C | NP_006155.2:p.Glu79Gln | 2/5 |  |  |
| **3091** | chr17 | 40481621 | T | C | 21 | STAT3 | XM_005257613.1 |  |  | missense_variant | XM_005257613.1:c.1469A>G | XP_005257670.1:p.Asn490Ser | 13/23 |  |  |
| **3091** | chr17 | 7577545 | T | C | 31 | TP53 | NM_000546.5 | 0 | probably_damaging | missense_variant | NM_000546.5:c.736A>G | NP_000537.3:p.Met246Val | 7/11 |  | likely_pathogenic |
| **3092** | chr19 | 15290236 | G | T | 72 | NOTCH3 | NM_000435.2 | 0 | possibly_damaging | missense_variant | NM_000435.2:c.3399C>A | NP_000426.2:p.His1133Gln | 21/33 |  |  |
| **3092** | chr17 | 7578211 | C | A | 35 | TP53 | NM_000546.5 | 0 | probably_damaging | missense_variant | NM_000546.5:c.638G>T | NP_000537.3:p.Arg213Leu | 6/11 |  |  |
| **3093** | chr17 | 7578536 | T | A | 53 | TP53 | NM_000546.5 |  |  | stop_gained | NM_000546.5:c.394A>T | NP_000537.3:p.Lys132Ter | 5/11 |  |  |
| **3093** | chr16 | 2138295 | G | C | 34 | TSC2 | XM_005255527.1 |  |  | missense_variant | XM_005255527.1:c.5501G>C | XP_005255584.1:p.Arg1834Pro | 41/42 |  |  |
| **3094** | chr8 | 61778097 | G | C | 50 | CHD7 | NM_017780.3 |  | benign | missense_variant | NM_017780.3:c.8599G>C | NP_060250.2:p.Ala2867Pro | 38/38 |  |  |
| **3094** | chr1 | 162741850 | G | T | 16 | DDR2 | XM_005245220.1 |  |  | missense_variant | XM_005245220.1:c.1586G>T | XP_005245277.1:p.Gly529Val | 11/16 |  |  |
| **3094** | chr13 | 48916852 | T | A | 30 | RB1 | NM_000321.2 |  |  | splice_donor_variant | NM_000321.2:c.380+2T>A |  |  | 3/26 |  |

**Supplementary Table S3.** Coverage statistics for each sample.
