# Supplementary material for: PI3K/mTORC2-RICTOR axis in early squamous non-small-cell lung cancer: genomics, molecular expression, and clinical relevance
Source: Ther Adv Med Oncol. 2025 Nov 7;17:17588359251370510. doi: 10.1177/17588359251370510 (PMC12597913; doi:10.1177/17588359251370510)
Supplement: sj-docx-5-tam-10.1177_17588359251370510 – Supplemental material for PI3K/mTORC2-RICTOR axis in early squamous non-small-cell lung cancer: genomics, molecular expression, and clinical relevance [file sj-docx-5-tam-10.1177_17588359251370510.docx]

**Supplementary Table S4.** Clinical and pathological characteristics of the 409 patients included in the TCGA set, separated in prognostic groups (PP and GP) according to clinicopathological information.

| **TCGA Set** | **PP**  **(N = 195)** | **GP**  **(N = 214)** |
| --- | --- | --- |
|  | Patient number (%) | |
| Median age [years]  *Range* | 67  [39 - 84] | 69  [40 - 85] |
| Gender |  |  |
| *Male* | 144 (73.8) | 155 (72.4) |
| *Female* | 51 (26.2) | 59 (27.6) |
| TNM Staging |  |  |
| *I* | 0 (0.0) | 196 (91.6) |
| *II* | 117 (60.0) | 18 (9.2) |
| *III* | 78 (40.0) | 0 (0.0) |
| Lymph nodes |  |  |
| *Negative* | 43 (22.1) | 214 (100.0) |
| *Positive* | 152 (77.9) | 0 (0.0) |
| Tumor size  [T descriptor according to  TNM 7th edition] |  |  |
| *1* | 18 (9.2) | 69 (32.2) |
| *2* | 96 (49.2) | 145 (67.8) |
| *3* | 61 (31.3) | 0 (0.0) |
| *4* | 20 (10.3) | 0 (0.0) |
| Node status  [N descriptor according to  TNM 7th edition] |  |  |
| *0* | 43 (22.1) | 214 (0.0) |
| *1* | 111 (56.9) | 0 (0.0) |
| *2* | 36 (18.5) | 0 (0.0) |
| *3* | 5 (2.6) | 0 (0.0) |

**Legend.** PP, poor prognosis; GP, good prognosis; N, number.
