# Supplementary material for: PI3K/mTORC2-RICTOR axis in early squamous non-small-cell lung cancer: genomics, molecular expression, and clinical relevance
Source: Ther Adv Med Oncol. 2025 Nov 7;17:17588359251370510. doi: 10.1177/17588359251370510 (PMC12597913; doi:10.1177/17588359251370510)
Supplement: sj-docx-6-tam-10.1177_17588359251370510 – Supplemental material for PI3K/mTORC2-RICTOR axis in early squamous non-small-cell lung cancer: genomics, molecular expression, and clinical relevance [file sj-docx-6-tam-10.1177_17588359251370510.docx]

**Supplementary Table S5.** Prevalence of somatic mutations reported for 38 genes in 409 patients of the TCGA set, according to the prognostic groups (PP and GP). The prevalence as detected in the training set is shown for reference.

| **Gene** | **PP N** | **PP %** | **GP N** | **GP %** | **Total N** | **Total % TCGA** | **Total %  Training set** |
| --- | --- | --- | --- | --- | --- | --- | --- |
| ***ALK*** | 7 | 3.6 | 9 | 4.2 | 16 | 3.9 | 3.3 |
| ***APC*** | 13 | 6.7 | 9 | 4.2 | 22 | 5.4 | 1.7 |
| ***ARID1A*** | 12 | 6.2 | 14 | 6.5 | 26 | 6.4 | 3.3 |
| ***ARID2*** | 10 | 5.1 | 7 | 3.3 | 17 | 4.2 | 1.7 |
| ***ATM*** | 16 | 8.2 | 8 | 3.7 | 24 | 5.9 | 3.3 |
| ***BAP1*** | 2 | 1.0 | 1 | 0.5 | 3 | 0.7 | 5.0 |
| ***CDH1*** | 5 | 2.6 | 1 | 0.5 | 6 | 1.5 | 6.7 |
| ***CDH10*** | 38 | 19.5 | 45 | 21.0 | 83 | 20.3 | 8.3 |
| ***CDKN2A*** | 31 | 15.9 | 29 | 13.6 | 60 | 14.7 | 6.7 |
| ***CHD7*** | 17 | 8.7 | 15 | 7.0 | 32 | 7.8 | 3.3 |
| ***CUL3*** | 12 | 6.2 | 8 | 3.7 | 20 | 4.9 | 3.3 |
| ***DDR2*** | 10 | 5.1 | 3 | 1.4 | 13 | 3.2 | 5.0 |
| ***EGFR*** | 5 | 2.6 | 7 | 3.3 | 12 | 2.9 | 1.7 |
| ***FBXW7*** | 12 | 6.2 | 5 | 2.3 | 17 | 4.2 | 3.3 |
| ***FLT3*** | 8 | 4.1 | 3 | 1.4 | 11 | 2.7 | 1.7 |
| ***KAT6A*** | 6 | 3.1 | 13 | 6.1 | 19 | 4.6 | 1.7 |
| ***KDM6A*** | 15 | 7.7 | 4 | 1.9 | 19 | 4.6 | 3.3 |
| ***KEAP1*** | 19 | 9.7 | 19 | 8.9 | 38 | 9.3 | 10.0 |
| ***KMT2D*** | 44 | 22.6 | 48 | 22.4 | 92 | 22.5 | 16.7 |
| ***KRAS*** | 5 | 2.6 | 1 | 0.5 | 6 | 1.5 | 5.0 |
| ***NF1*** | 28 | 14.4 | 20 | 9.3 | 48 | 11.7 | 3.3 |
| ***NFE2L2*** | 28 | 14.4 | 36 | 16.8 | 64 | 15.6 | 6.7 |
| ***NOTCH1*** | 17 | 8.7 | 20 | 9.3 | 37 | 9.0 | 3.3 |
| ***NOTCH2*** | 7 | 3.6 | 13 | 6.1 | 20 | 4.9 | 1.7 |
| ***NOTCH3*** | 10 | 5.1 | 4 | 1.9 | 14 | 3.4 | 5.0 |
| ***NRAS*** | 2 | 1.0 | 3 | 1.4 | 5 | 1.2 | 1.7 |
| ***PAPPA2*** | 35 | 17.9 | 46 | 21.5 | 81 | 19.8 | 3.3 |
| ***PIK3CA*** | 23 | 11.8 | 23 | 10.7 | 46 | 11.2 | 5.0 |
| ***PTEN*** | 13 | 6.7 | 29 | 13.6 | 42 | 10.3 | 10.0 |
| ***RASA1*** | 12 | 6.2 | 14 | 6.5 | 26 | 6.4 | 1.7 |
| ***RB1*** | 14 | 7.2 | 12 | 5.6 | 26 | 6.4 | 10.0 |
| ***SMAD4*** | 3 | 1.5 | 4 | 1.9 | 7 | 1.7 | 1.7 |
| ***SMARCA4*** | 10 | 5.1 | 6 | 2.8 | 16 | 3.9 | 1.7 |
| ***STAT3*** | 0 | 0.0 | 3 | 1.4 | 3 | 0.7 | 1.7 |
| ***TIE1*** | 6 | 3.1 | 8 | 3.7 | 14 | 3.4 | 6.7 |
| ***TP53*** | 159 | 81.5 | 181 | 84.6 | 340 | 83.1 | 88.3 |
| ***TSC1*** | 2 | 1.0 | 6 | 2.8 | 8 | 2.0 | 5.0 |
| ***TSC2*** | 7 | 3.6 | 6 | 2.8 | 13 | 3.2 | 3.3 |

Legend - N, number of mutated cases; PP, poor prognosis; GP, good prognosis.
