# Supplementary material for: PI3K/mTORC2-RICTOR axis in early squamous non-small-cell lung cancer: genomics, molecular expression, and clinical relevance
Source: Ther Adv Med Oncol. 2025 Nov 7;17:17588359251370510. doi: 10.1177/17588359251370510 (PMC12597913; doi:10.1177/17588359251370510)
Supplement: sj-docx-7-tam-10.1177_17588359251370510 – Supplemental material for PI3K/mTORC2-RICTOR axis in early squamous non-small-cell lung cancer: genomics, molecular expression, and clinical relevance [file sj-docx-7-tam-10.1177_17588359251370510.docx]

**Supplementary Table S6.** Prevalence of copy number variations in 409 patients of the TCGA set, according to the prognostic groups (PP and GP). The prevalence as detected in the training set (60 cases) is shown for reference. Panel A shows the analysis for “strong” TCGA CNV; Panel B shows the analysis for “shallow” TCGA CNV.

| **Panel A - Strong TCGA CNV** | | | | | | | |
| --- | --- | --- | --- | --- | --- | --- | --- |
| **Gene [CNV type]** | **PP N** | **PP %** | **GP N** | **GP %** | **Total N** | **Total % TCGA** | **Total %  Training set** |
| AKT1 [Gain] | 7 | 3.6 | 5 | 2.3 | 12 | 2.9 | 8.3 |
| APC [Loss] | 1 | 0.5 | 2 | 0.9 | 3 | 0.7 | 10 |
| BCL2L1 [Gain] | 8 | 4.1 | 12 | 5.6 | 20 | 4.9 | 18.3 |
| CCND1 [Gain] | 29 | 14.9 | 26 | 12.1 | 55 | 13.4 | 15 |
| CCND2 [Gain] | 4 | 2.1 | 4 | 1.9 | 8 | 2.0 | 20 |
| CDKN2A [Loss] | 54 | 27.7 | 53 | 24.8 | 107 | 26.2 | 26.7 |
| DDR2 [Gain] | 9 | 4.6 | 8 | 3.7 | 17 | 4.2 | 10 |
| EGFR [Gain] | 16 | 8.2 | 10 | 4.7 | 26 | 6.4 | 13.3 |
| ERBB2 [Gain] | 6 | 3.1 | 3 | 1.4 | 9 | 2.2 | 13.3 |
| FGFR1 [Gain] | 34 | 17.4 | 36 | 16.8 | 70 | 17.1 | 18.3 |
| FGFR2 [Gain] | 0 | 0.0 | 1 | 0.5 | 1 | 0.2 | 11.7 |
| FGFR3 [Gain] | 1 | 0.5 | 0 | 0.0 | 1 | 0.2 | 8.3 |
| FOXP1 [Loss] | 15 | 7.7 | 10 | 4.7 | 25 | 6.1 | 28.3 |
| FOXP4 [Loss] | 0 | 0.0 | 1 | 0.5 | 1 | 0.2 | 10 |
| FRS2 [Gain] | 4 | 2.1 | 9 | 4.2 | 13 | 3.2 | 20 |
| JAK3 [Gain] | 2 | 1.0 | 2 | 0.9 | 4 | 1.0 | 20 |
| KIT [Gain] | 9 | 4.6 | 11 | 5.1 | 20 | 4.9 | 8.3 |
| MCL1 [Gain] | 11 | 5.6 | 8 | 3.7 | 19 | 4.6 | 23.3 |
| MDM2 [Gain] | 3 | 1.5 | 10 | 4.7 | 13 | 3.2 | 20 |
| MET [Gain] | 3 | 1.5 | 2 | 0.9 | 5 | 1.2 | 5 |
| MYC [Gain] | 19 | 9.7 | 15 | 7.0 | 34 | 8.3 | 6.7 |
| MYCL1 [Gain] | 3 | 1.5 | 5 | 2.3 | 8 | 2.0 | 6.7 |
| MYCN [Gain] | 6 | 3.1 | 1 | 0.5 | 7 | 1.7 | 6.7 |
| NFE2L2 [Gain] | 5 | 2.6 | 11 | 5.1 | 16 | 3.9 | 11.7 |
| NOTCH1 [Loss] | 2 | 1.0 | 0 | 0.0 | 2 | 0.5 | 8.3 |
| PBRM1 [Loss] | 2 | 1.0 | 3 | 1.4 | 5 | 1.2 | 23.3 |
| PDGFRA [Gain] | 9 | 4.6 | 10 | 4.7 | 19 | 4.6 | 16.7 |
| PIK3CA [Gain] | 76 | 39.0 | 81 | 37.9 | 157 | 38.4 | 56.7 |
| PTCH1 [Loss] | 4 | 2.1 | 0 | 0.0 | 4 | 1.0 | 11.7 |
| PTEN [Loss] | 26 | 13.3 | 17 | 7.9 | 43 | 10.5 | 35 |
| RB1 [Loss] | 7 | 3.6 | 7 | 3.3 | 14 | 3.4 | 25 |
| RICTOR [Gain] | 19 | 9.7 | 24 | 11.2 | 43 | 10.5 | 23.3 |
| SMAD4 [Loss] | 5 | 2.6 | 3 | 1.4 | 8 | 2.0 | 21.7 |
| SMARCB1 [Gain] | 6 | 3.1 | 1 | 0.5 | 7 | 1.7 | 25 |
| SOX2 [Gain] | 78 | 40.0 | 88 | 41.1 | 166 | 40.6 | 78.3 |
| TERT [Gain] | 26 | 13.3 | 33 | 15.4 | 59 | 14.4 | 15 |
| TET2 [Loss] | 0 | 0.0 | 0 | 0.0 | 0 | 0.0 | 6.7 |
| TP53 [Loss] | 1 | 0.5 | 1 | 0.5 | 2 | 0.5 | 6.7 |
| TP63 [Gain] | 61 | 31.3 | 71 | 33.2 | 132 | 32.3 | 40 |
| TSC2 [Gain] | 0 | 0.0 | 0 | 0.0 | 0 | 0.0 | 1.7 |

**Legend -** N, number of mutated cases; PP, poor prognosis; GP, good prognosis.

| **Panel B - Shallow TCGA CNV** | | | | | | | |
| --- | --- | --- | --- | --- | --- | --- | --- |
| **Gene [CNV type]** | **PP N** | **PP %** | **GP N** | **GP %** | **Total N** | **Total % TCGA** | **Total %  Training set** |
| AKT1 [Gain] | 65 | 33.3 | 61 | 28.5 | 126 | 30.8 | 8.3 |
| APC [Loss] | 151 | 77.4 | 167 | 78.0 | 318 | 77.8 | 10 |
| BCL2L1 [Gain] | 118 | 60.5 | 127 | 59.3 | 245 | 59.9 | 18.3 |
| CCND1 [Gain] | 72 | 36.9 | 76 | 35.5 | 148 | 36.2 | 15 |
| CCND2 [Gain] | 105 | 53.8 | 106 | 49.5 | 211 | 51.6 | 20 |
| CDKN2A [Loss] | 154 | 79.0 | 171 | 79.9 | 325 | 79.5 | 26.7 |
| DDR2 [Gain] | 107 | 54.9 | 111 | 51.9 | 218 | 53.3 | 10 |
| EGFR [Gain] | 105 | 53.8 | 99 | 46.3 | 204 | 49.9 | 13.3 |
| ERBB2 [Gain] | 71 | 36.4 | 66 | 30.8 | 137 | 33.5 | 13.3 |
| FGFR1 [Gain] | 84 | 43.1 | 101 | 47.2 | 185 | 45.2 | 18.3 |
| FGFR2 [Gain] | 19 | 9.7 | 24 | 11.2 | 43 | 10.5 | 11.7 |
| FGFR3 [Gain] | 15 | 7.7 | 19 | 8.9 | 34 | 8.3 | 8.3 |
| FOXP1 [Loss] | 170 | 87.2 | 184 | 86.0 | 354 | 86.6 | 28.3 |
| FOXP4 [Loss] | 44 | 22.6 | 49 | 22.9 | 93 | 22.7 | 10 |
| FRS2 [Gain] | 72 | 36.9 | 69 | 32.2 | 141 | 34.5 | 20 |
| JAK3 [Gain] | 58 | 29.7 | 46 | 21.5 | 104 | 25.4 | 20 |
| KIT [Gain] | 47 | 24.1 | 47 | 22.0 | 94 | 23.0 | 8.3 |
| MCL1 [Gain] | 107 | 54.9 | 107 | 50.0 | 214 | 52.3 | 23.3 |
| MDM2 [Gain] | 72 | 36.9 | 69 | 32.2 | 141 | 34.5 | 20 |
| MET [Gain] | 94 | 48.2 | 85 | 39.7 | 179 | 43.8 | 5 |
| MYC [Gain] | 143 | 73.3 | 139 | 65.0 | 282 | 68.9 | 6.7 |
| MYCL1 [Gain] | 29 | 14.9 | 36 | 16.8 | 65 | 15.9 | 6.7 |
| MYCN [Gain] | 109 | 55.9 | 94 | 43.9 | 203 | 49.6 | 6.7 |
| NFE2L2 [Gain] | 68 | 34.9 | 76 | 35.5 | 144 | 35.2 | 11.7 |
| NOTCH1 [Loss] | 89 | 45.6 | 100 | 46.7 | 189 | 46.2 | 8.3 |
| PBRM1 [Loss] | 170 | 87.2 | 186 | 86.9 | 356 | 87.0 | 23.3 |
| PDGFRA [Gain] | 46 | 23.6 | 48 | 22.4 | 94 | 23.0 | 16.7 |
| PIK3CA [Gain] | 174 | 89.2 | 197 | 92.1 | 371 | 90.7 | 56.7 |
| PTCH1 [Loss] | 87 | 44.6 | 99 | 46.3 | 186 | 45.5 | 11.7 |
| PTEN [Loss] | 101 | 51.8 | 124 | 57.9 | 225 | 55.0 | 35 |
| RB1 [Loss] | 137 | 70.3 | 146 | 68.2 | 283 | 69.2 | 25 |
| RICTOR [Gain] | 146 | 74.9 | 159 | 74.3 | 305 | 74.6 | 23.3 |
| SMAD4 [Loss] | 88 | 45.1 | 81 | 37.9 | 169 | 41.3 | 21.7 |
| SMARCB1 [Gain] | 88 | 45.1 | 113 | 52.8 | 201 | 49.1 | 25 |
| SOX2 [Gain] | 177 | 90.8 | 197 | 92.1 | 374 | 91.4 | 78.3 |
| TERT [Gain] | 148 | 75.9 | 171 | 79.9 | 319 | 78.0 | 15 |
| TET2 [Loss] | 106 | 54.4 | 138 | 64.5 | 244 | 59.7 | 6.7 |
| TP53 [Loss] | 127 | 65.1 | 134 | 62.6 | 261 | 63.8 | 6.7 |
| TP63 [Gain] | 173 | 88.7 | 192 | 89.7 | 365 | 89.2 | 40 |
| TSC2 [Gain] | 35 | 17.9 | 31 | 14.5 | 66 | 16.1 | 1.7 |

**Legend -** N, number of mutated cases; PP, poor prognosis; GP, good prognosis.
