# Supplementary figures and images for: PI3K/mTORC2-RICTOR axis in early squamous non-small-cell lung cancer: genomics, molecular expression, and clinical relevance
Source: Ther Adv Med Oncol. 2025 Nov 7;17:17588359251370510. doi: 10.1177/17588359251370510 (PMC12597913; doi:10.1177/17588359251370510)

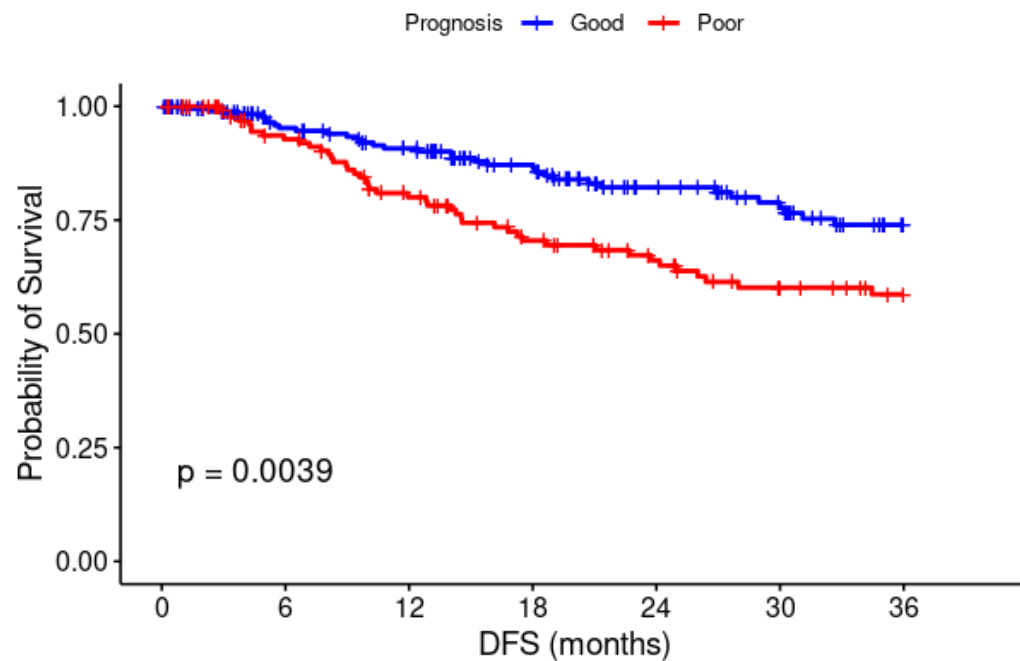

Prognosis

| Number at risk |     |     |     |     |    |    |    |
|----------------|-----|-----|-----|-----|----|----|----|
|                | 0   | 6   | 12  | 18  | 24 | 30 | 36 |
| Good           | 198 | 153 | 137 | 111 | 83 | 67 | 47 |
| Poor           | 142 | 112 | 89  | 71  | 58 | 47 | 39 |

DFS (months)

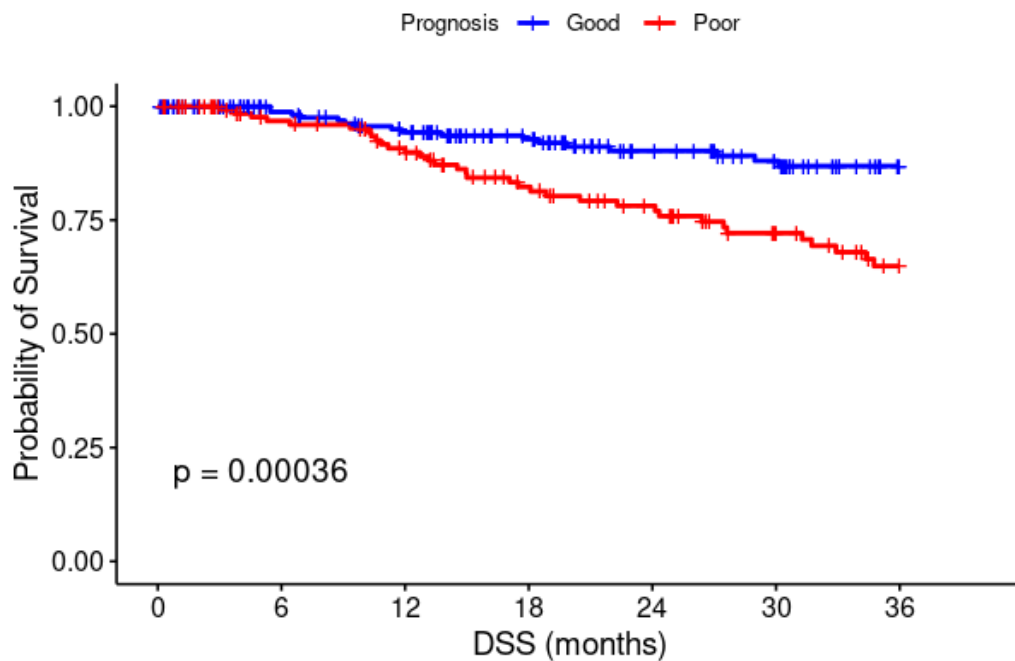

Prognosis

| Number at risk |     |     |     |     |    |    |    |
|----------------|-----|-----|-----|-----|----|----|----|
|                | 0   | 6   | 12  | 18  | 24 | 30 | 36 |
| Good           | 198 | 159 | 143 | 117 | 92 | 77 | 56 |
| Poor           | 142 | 117 | 101 | 82  | 69 | 54 | 41 |

DSS (months)

Supplement: sj-pdf-10-tam-10.1177_17588359251370510 – Supplemental material for PI3K/mTORC2-RICTOR axis in early squamous non-small-cell lung cancer: genomics, molecular expression, and clinical relevance [file sj-pdf-10-tam-10.1177_17588359251370510.pdf]

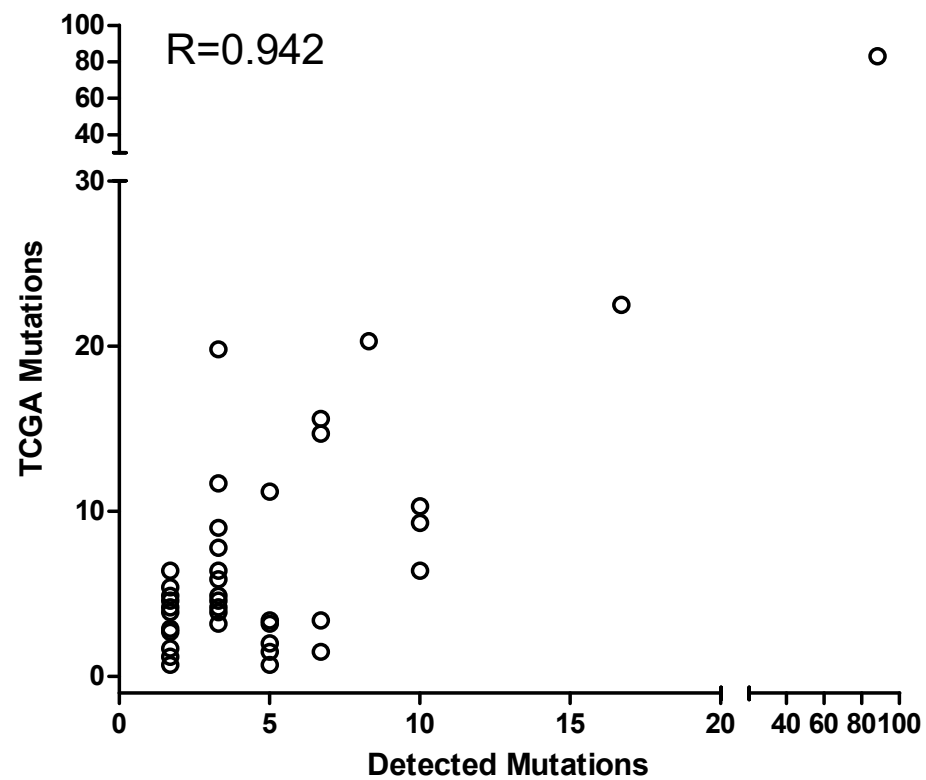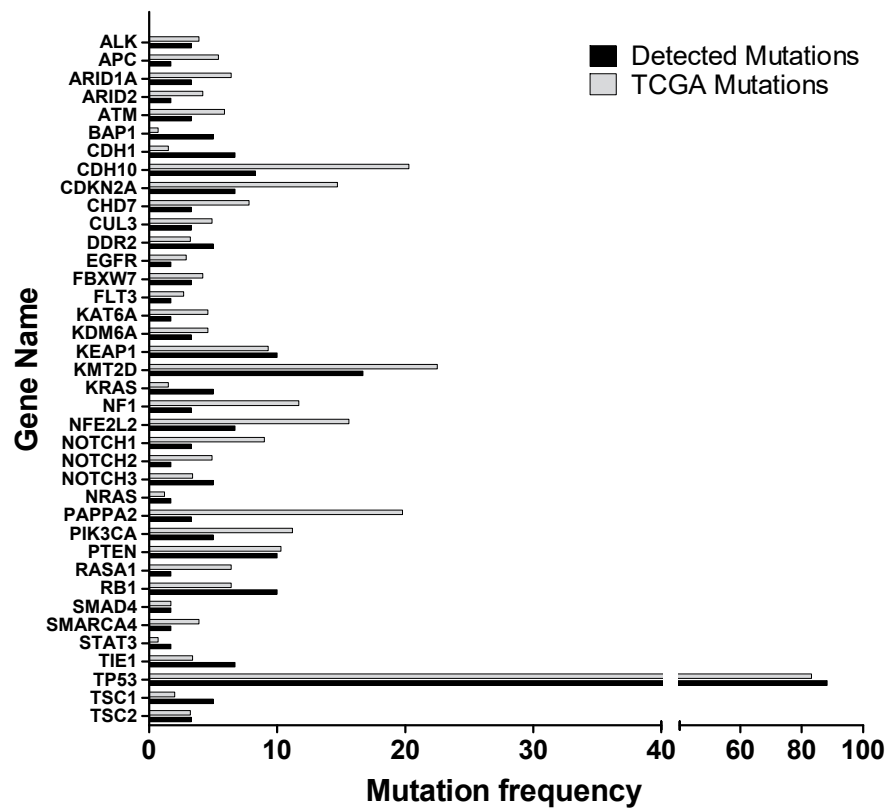

Supplement: sj-pdf-11-tam-10.1177_17588359251370510 – Supplemental material for PI3K/mTORC2-RICTOR axis in early squamous non-small-cell lung cancer: genomics, molecular expression, and clinical relevance [file sj-pdf-11-tam-10.1177_17588359251370510.pdf]

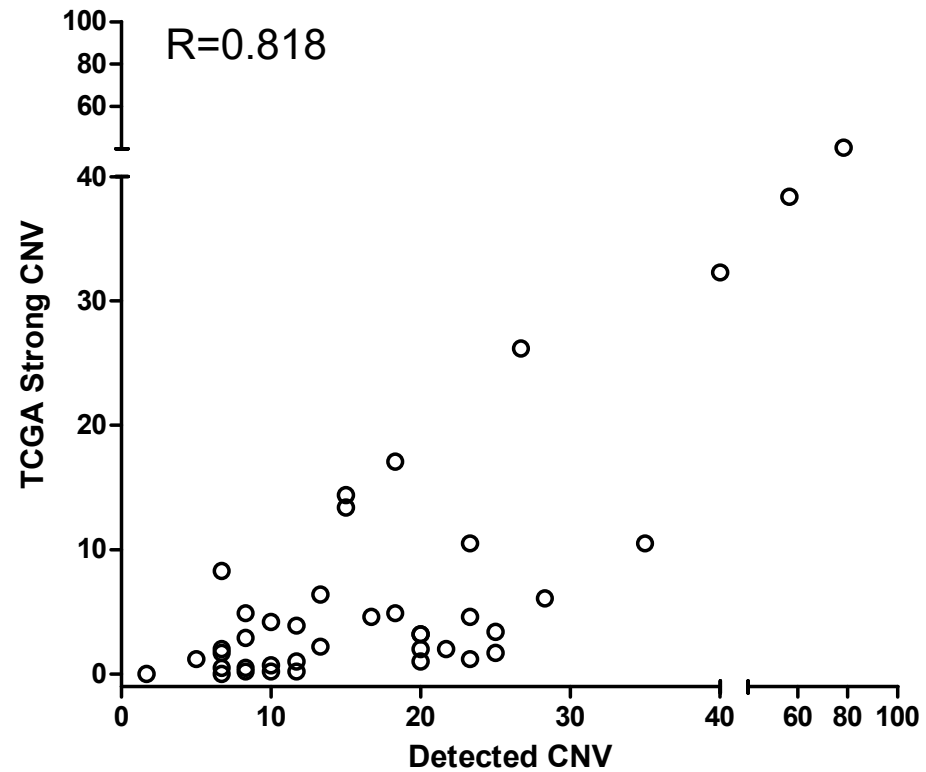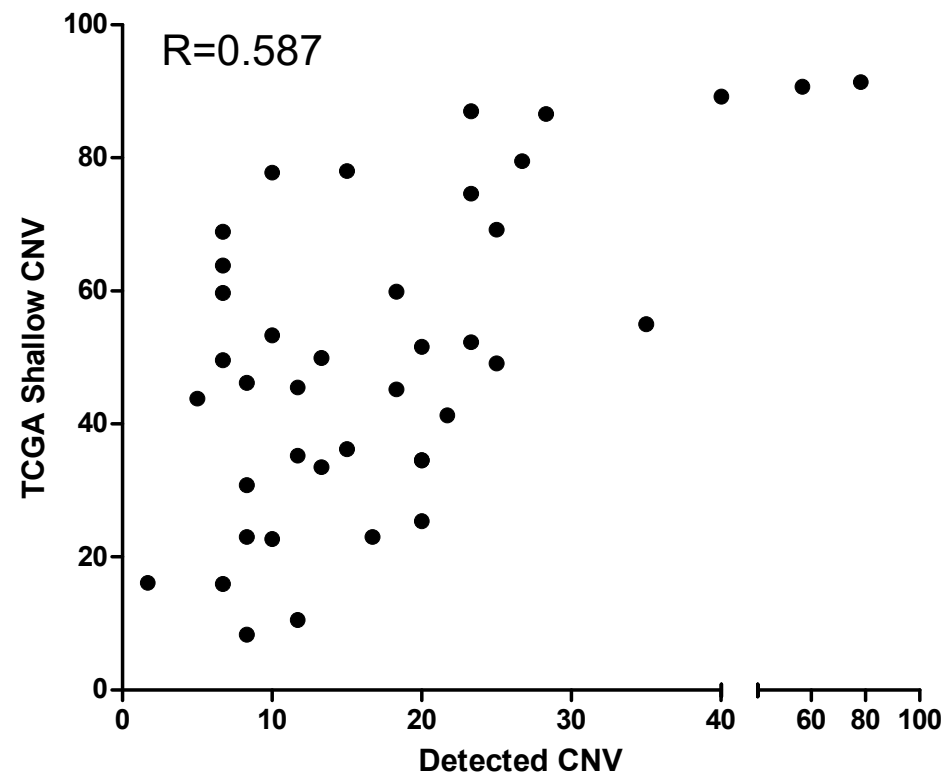

Supplement: sj-pdf-12-tam-10.1177_17588359251370510 – Supplemental material for PI3K/mTORC2-RICTOR axis in early squamous non-small-cell lung cancer: genomics, molecular expression, and clinical relevance [file sj-pdf-12-tam-10.1177_17588359251370510.pdf]

## Good Prognosis

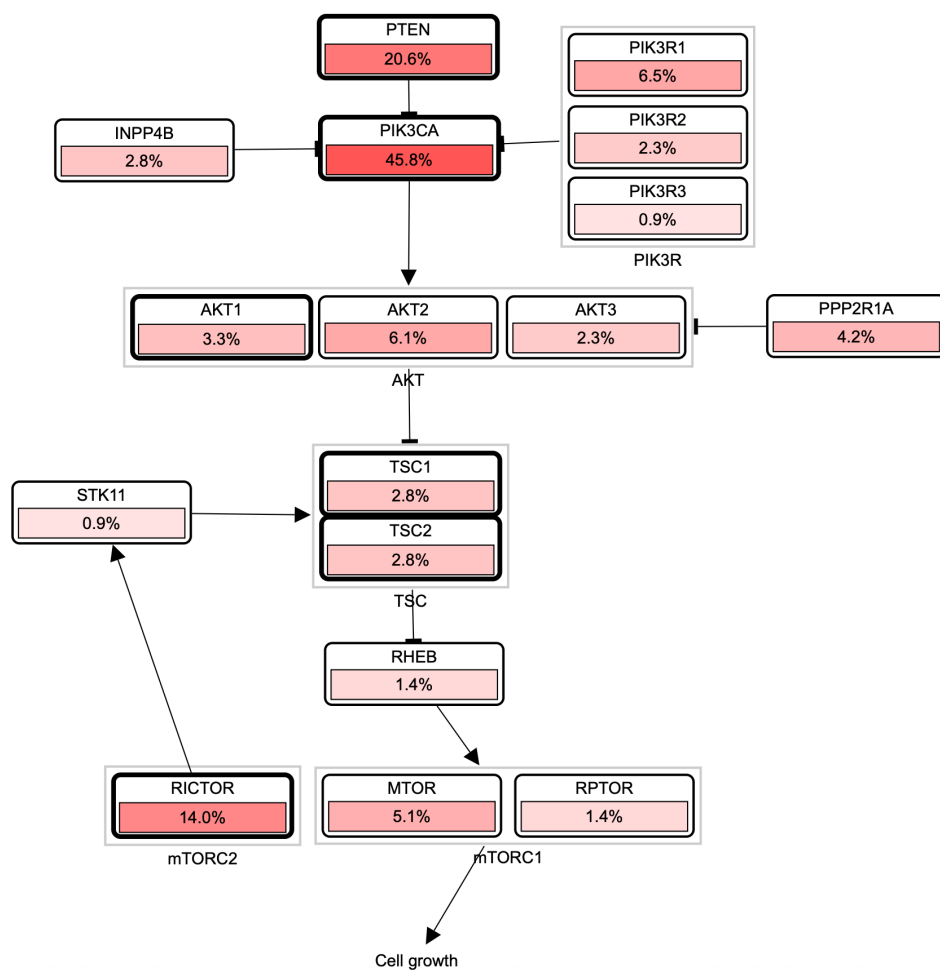

## Poor Prognosis

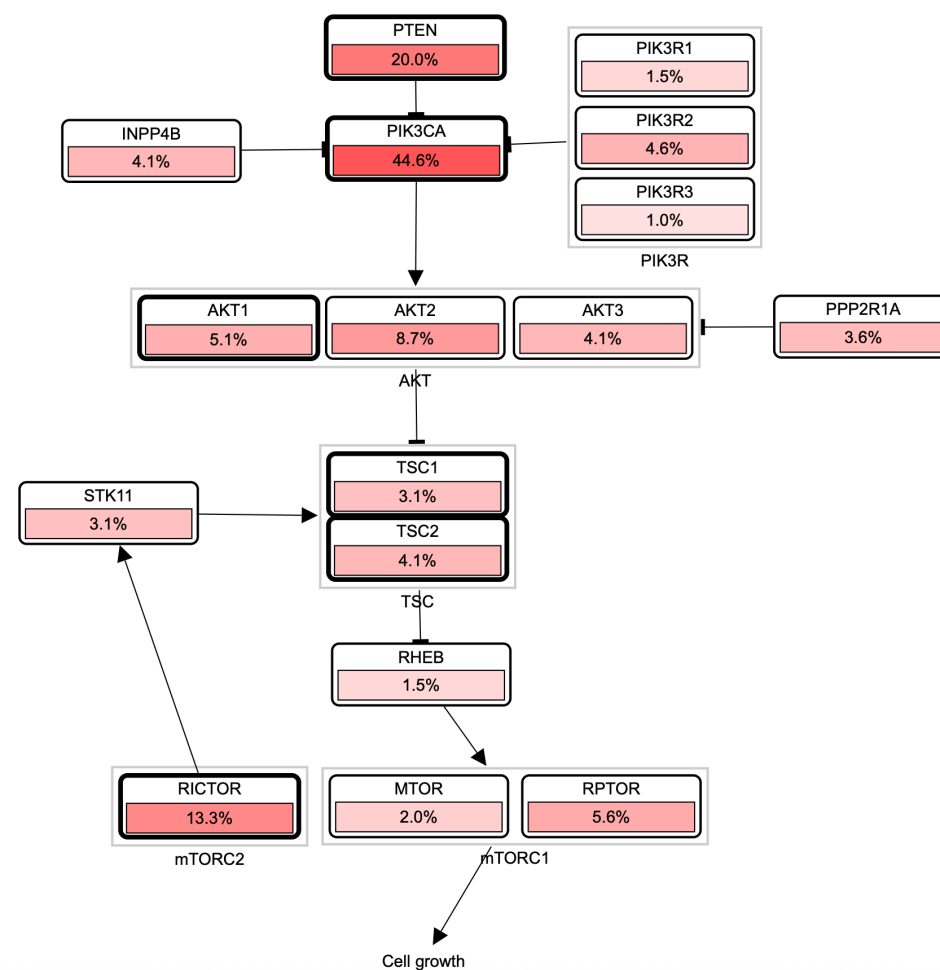

Supplement: sj-pdf-13-tam-10.1177_17588359251370510 – Supplemental material for PI3K/mTORC2-RICTOR axis in early squamous non-small-cell lung cancer: genomics, molecular expression, and clinical relevance [file sj-pdf-13-tam-10.1177_17588359251370510.pdf]

A]

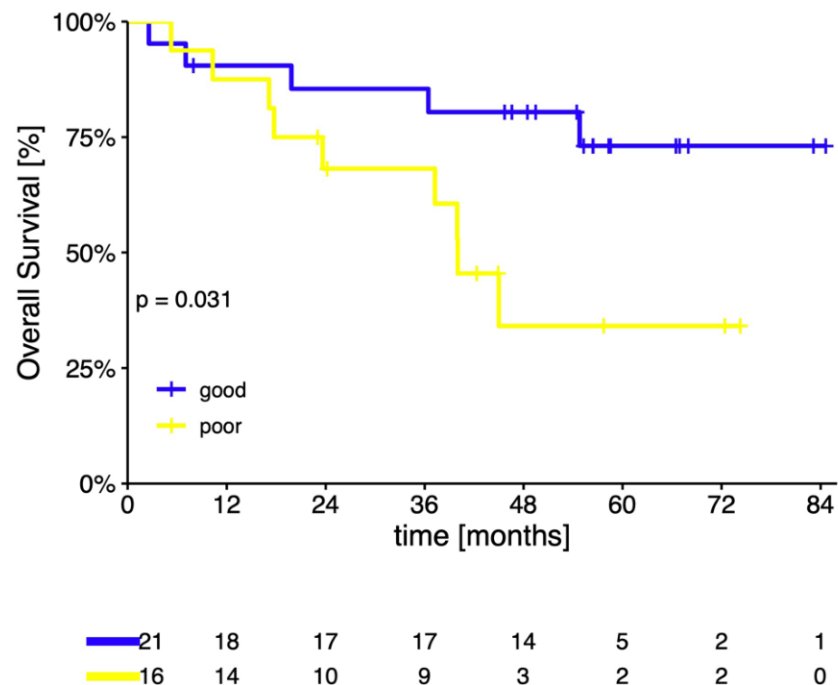

B]

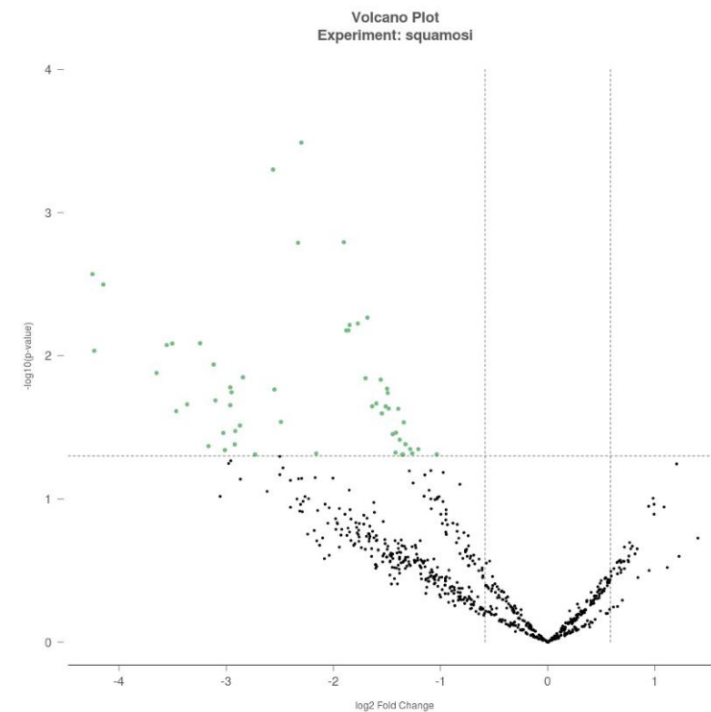

C]

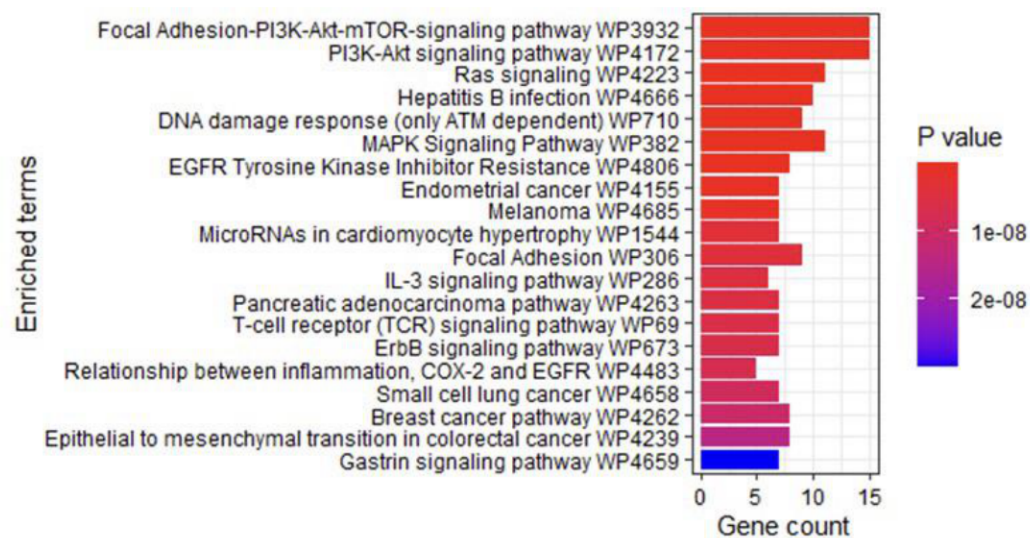

D]

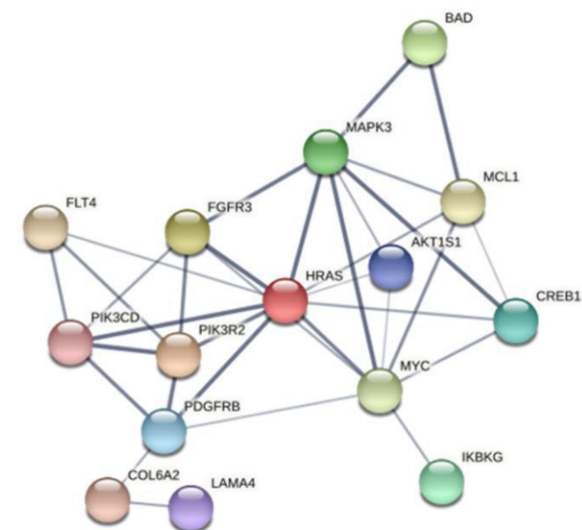

Supplement: sj-pdf-14-tam-10.1177_17588359251370510 – Supplemental material for PI3K/mTORC2-RICTOR axis in early squamous non-small-cell lung cancer: genomics, molecular expression, and clinical relevance [file sj-pdf-14-tam-10.1177_17588359251370510.pdf]

POOR

GOOD

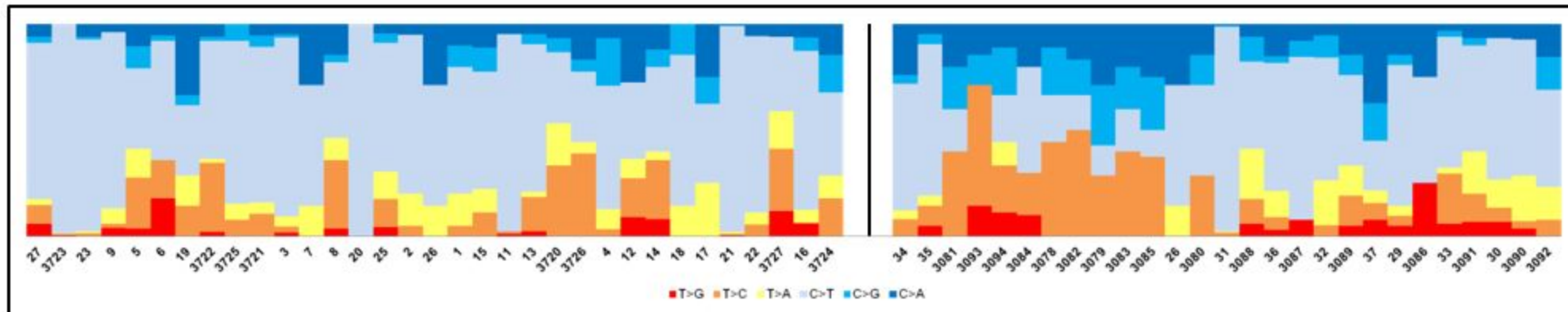

Mutations/Mb

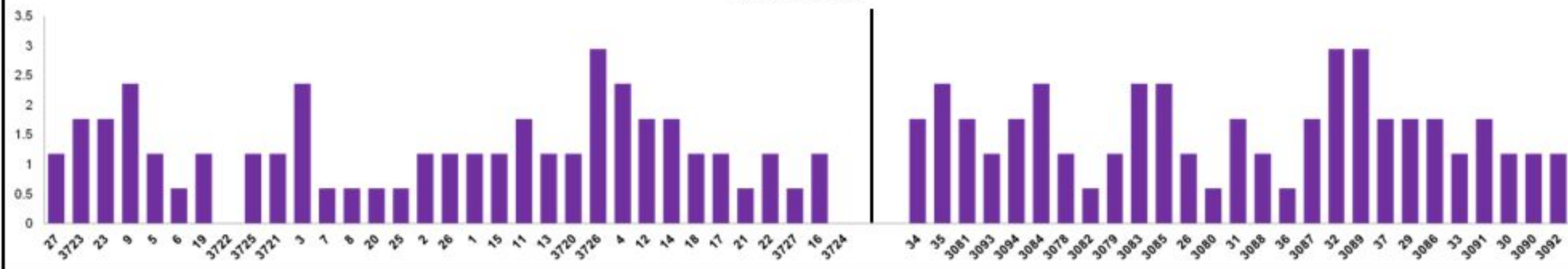

Indels/Mb

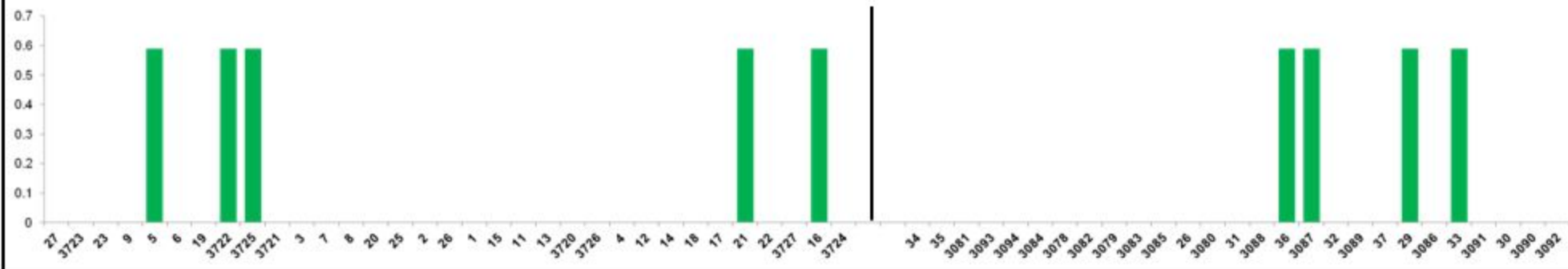

Supplement: sj-pdf-9-tam-10.1177_17588359251370510 – Supplemental material for PI3K/mTORC2-RICTOR axis in early squamous non-small-cell lung cancer: genomics, molecular expression, and clinical relevance [file sj-pdf-9-tam-10.1177_17588359251370510.pdf]
