# Supplementary material for: PI3K/mTORC2-RICTOR axis in early squamous non-small-cell lung cancer: genomics, molecular expression, and clinical relevance
Source: Ther Adv Med Oncol. 2025 Nov 7;17:17588359251370510. doi: 10.1177/17588359251370510 (PMC12597913; doi:10.1177/17588359251370510)
Supplement: sj-pdf-8-tam-10.1177_17588359251370510 – Supplemental material for PI3K/mTORC2-RICTOR axis in early squamous non-small-cell lung cancer: genomics, molecular expression, and clinical relevance [file sj-pdf-8-tam-10.1177_17588359251370510.pdf]

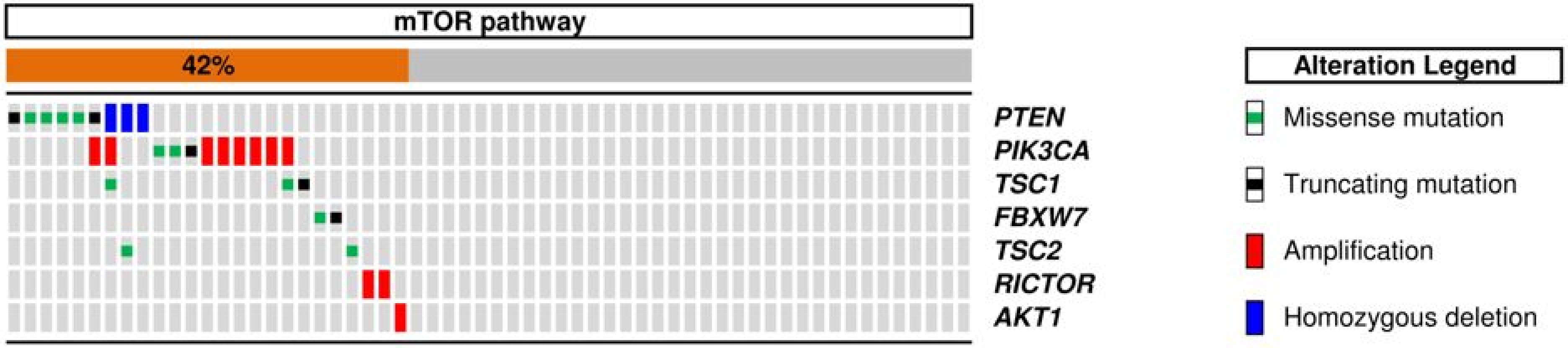
mTOR pathway
42%
PTEN
PIK3CA
TSC1
FBXW7
TSC2
RICTOR
AKT1
Alteration Legend
Missense mutation
Truncating mutation
Amplification
Homozygous deletion
